# Construction and experimental validation of a novel ferroptosis‐related gene signature for myelodysplastic syndromes

**DOI:** 10.1002/iid3.1221

**Published:** 2024-04-05

**Authors:** Yidong Zhu, Jun He, Rong Wei, Jun Liu

**Affiliations:** ^1^ Department of Traditional Chinese Medicine, Shanghai Tenth People's Hospital Tongji University School of Medicine Shanghai China; ^2^ Department of Hematology, Shanghai Tenth People's Hospital Tongji University School of Medicine Shanghai China

**Keywords:** ferroptosis, gene signature, immunity, machine learning, myelodysplastic syndromes

## Abstract

**Background:**

Myelodysplastic syndromes (MDS) are clonal hematopoietic disorders characterized by morphological abnormalities and peripheral blood cytopenias, carrying a risk of progression to acute myeloid leukemia. Although ferroptosis is a promising target for MDS treatment, the specific roles of ferroptosis‐related genes (FRGs) in MDS diagnosis have not been elucidated.

**Methods:**

MDS‐related microarray data were obtained from the Gene Expression Omnibus database. A comprehensive analysis of FRG expression levels in patients with MDS and controls was conducted, followed by the use of multiple machine learning methods to establish prediction models. The predictive ability of the optimal model was evaluated using nomogram analysis and an external data set. Functional analysis was applied to explore the underlying mechanisms. The mRNA levels of the model genes were verified in MDS clinical samples by quantitative real‐time polymerase chain reaction (qRT‐PCR).

**Results:**

The extreme gradient boosting model demonstrated the best performance, leading to the identification of a panel of six signature genes: *SREBF1*, *PTPN6*, *PARP9*, *MAP3K11*, *MDM4*, and *EZH2*. Receiver operating characteristic curves indicated that the model exhibited high accuracy in predicting MDS diagnosis, with area under the curve values of 0.989 and 0.962 for the training and validation cohorts, respectively. Functional analysis revealed significant associations between these genes and the infiltrating immune cells. The expression levels of these genes were successfully verified in MDS clinical samples.

**Conclusion:**

Our study is the first to identify a novel model using FRGs to predict the risk of developing MDS. FRGs may be implicated in MDS pathogenesis through immune‐related pathways. These findings highlight the intricate correlation between ferroptosis and MDS, offering insights that may aid in identifying potential therapeutic targets for this debilitating disorder.

## Graphical abstract

Our study represents the first identification of the involvement of ferroptosis‐related genes (FRGs) in myelodysplastic syndromes (MDS) and the establishment of a novel risk model based on six FRGs for assessing MDS development. These genes potentially contribute to MDS pathogenesis and progression through immune‐related pathways. The findings underscore the intricate correlation between ferroptosis and MDS, providing insights that could facilitate the identification of therapeutic targets for this disorder.

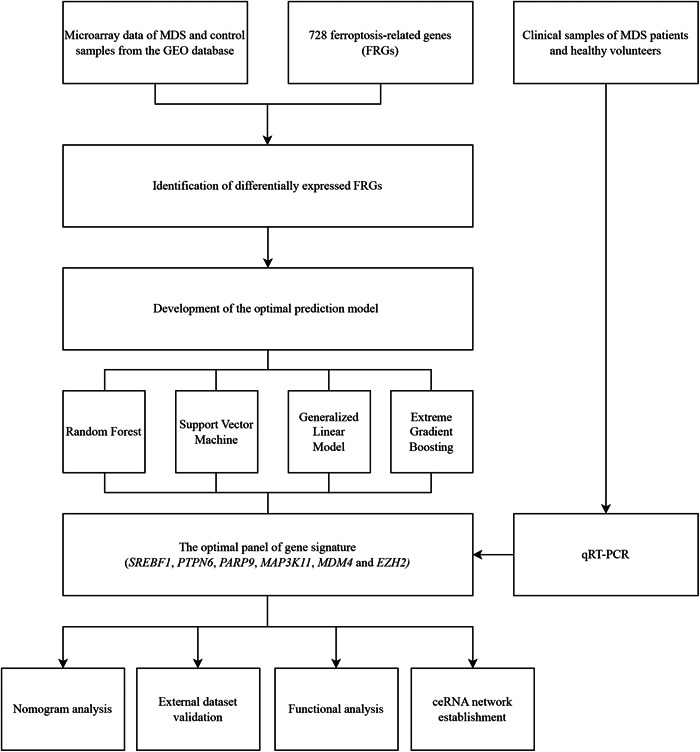

## 1 INTRODUCTION

Myelodysplastic syndromes (MDS) are a group of clonal hematopoietic diseases characterized by abnormal development of bone marrow cells, ineffective hematopoiesis, peripheral cytopenia, and a risk of transformation to acute myeloid leukemia.^1^ The clinical presentation is nonspecific and includes symptoms and signs of thrombocytopenia, anemia, and neutropenia.^2^ Diagnosis of MDS primarily relies on the presence of cytopenia, morphological evidence of dysplasia in bone marrow aspirate and biopsy, and clonal cytogenetic abnormalities.^1^, ^3^, ^4^ However, MDS cases vary in clinical presentation, with some exhibiting atypical features, including inapparent or absent cytopenia and dysplasia, upon examination.^5^, ^6^ Diagnostic discrepancies may occur in 12% of patients at the time of initial presentation, affecting therapeutic decision‐making.^7^ Timely and accurate diagnosis, followed by effective management, can significantly interfere with disease progression and improve overall prognosis.^8^ Therefore, the identification of novel and reliable biomarkers is imperative for improving diagnostic accuracy, which is essential for the effective treatment of MDS.

A considerable percentage of patients with MDS experience iron overload, which is primarily attributed to intermittent blood transfusions and ineffective hematopoiesis.^9^ In severe cases, this overload may trigger an excessive accumulation of reactive oxygen species, thereby inducing ferroptosis, a unique form of iron‐dependent cell death.^10^ Ferroptosis arises from a redox imbalance in the production of oxidants and antioxidants as a consequence of the abnormal expression and activity of several redox‐active enzymes involved in the production or detoxification of lipid oxidation products and free radicals.^11^, ^12^, ^13^, ^14^, ^15^ Increasing evidence indicates that ferroptosis plays a critical role in cancer progression.^16^, ^17^ For instance, Ubellacker et al. reported that lymphoid tissues protected tumor cells from ferroptosis and promoted melanoma metastasis.^18^ Nagpal et al. suggested that the induction of ferroptosis inhibited brain metastasis of tumors in a spontaneous mouse model of HER2‐positive breast cancer.^19^ Additionally, ferroptosis injury could trigger inflammation‐related immunosuppression in the tumor microenvironment, thereby favoring tumor growth.^20^ Given its distinct features, ferroptosis has attracted substantial interest as a potential treatment target for hematological malignancies, such as MDS, leukemia, lymphoma, and multiple myeloma.^21^ A recent study showed that the antileukemic drug decitabine induces ferroptosis in MDS. Treatment of MDS cell lines with decitabine leads to increased levels of reactive oxygen species and reduced activity of glutathione and glutathione peroxidase 4, suggesting a significant association between ferroptosis and MDS.^22^ However, the role of ferroptosis‐related genes (FRGs) in the pathogenesis of MDS remains unclear.

Studies have demonstrated that FRGs have the potential to predict immune responses and clinical outcomes in hematological malignancies, including lymphoma,^23^, ^24^, ^25^ leukemia,^26^, ^27^, ^28^ and multiple myeloma.^29^, ^30^, ^31^ In this study, we aimed to examine and validate the accuracy of FRGs as biomarkers for MDS. This study was initiated by performing differential expression analysis between MDS and control samples, followed by various machine learning methods to build a predictive model with differentially expressed FRGs. The efficacy of the model was evaluated using nomogram analysis and an external data set. Functional analysis was conducted to explore potential mechanisms. In addition, the mRNA levels of the identified genes in clinical MDS samples were confirmed by quantitative real‐time polymerase chain reaction (qRT‐PCR). This study is the first to establish an FRG diagnostic signature in patients with MDS, which will enhance our understanding of the pathogenesis and progression of MDS and aid in the development of personalized therapy strategies in clinical practice.

## 2 MATERIALS AND METHODS

### 2.1 Data collection

Microarray data and clinical information from patients with MDS and control samples were downloaded from the Gene Expression Omnibus database (GEO, https://www.ncbi.nlm.nih.gov/geo/). All data sets were normalized to remove batch effects. The GSE19429 data set, consisting of 183 MDS and 17 normal samples, was used as the training cohort. The GSE58831 data set, containing 159 MDS and 17 normal samples, was chosen as the validation cohort. In total, 728 FRGs were analyzed based on the FerrDb database (Supplementary Table S1).

### 2.2 Screening of differentially expressed FRGs

In the training cohort, the expression of FRGs in both MDS and control samples was extracted. Differential expression analysis was conducted using the “limma” package. FRGs with *p‐*values < 0.05 were retained.

### 2.3 Establishment of the optimal prediction model

Multiple machine learning methods were employed to develop a prediction model using the differentially expressed FRGs. Random forest (RF) is an ensemble of classification and regression trees that addresses overfitting and exhibits greater stability in high‐dimensional parameter spaces and in the presence of outliers.^32^, ^33^ Support vector machine (SVM) is a powerful method for building classifiers and creating decision boundaries between 2 classes to predict labels from one or more feature vectors.^34^, ^35^ Generalized linear models (GLMs) are statistical models that enable the modeling of relations between a response variable and one or more predictor variables, extending linear regression models to handle non‐normal distributions of the response variable, including binary, count, or continuous data with nonconstant variance.^36^, ^37^ Extreme gradient boosting (XGB) is a machine learning model that integrates multiple weak learners to achieve a stronger learning effect, exhibiting strong flexibility and scalability advantages.^38^, ^39^ The “Caret” package adjusts the parameters of these models through a grid search. These machine learning models were implemented with default parameters. Prediction models based on these algorithms were constructed accordingly. Subsequently, the residual distributions and feature importance of these models were analyzed. Receiver operating characteristic (ROC) curves were generated to evaluate the model accuracy and specificity. Based on the aforementioned performance, the optimal model was determined, and the top 6 feature variables in the model were selected as the optimal combination of gene signatures for MDS diagnosis. The results of these analyses were visualized using the “caret,” “DALEX,” “ggplot2,” “randomForest,” “kernlab,” “pROC,” and “xgboost” packages.

### 2.4 Nomogram construction

A nomogram was generated using the identified gene signature to predict the risk of MDS. We conducted nomogram analysis to evaluate the predictive ability of the model using the “rms” and “rmda” packages.

### 2.5 External data set validation

The expression levels of signature genes were verified in the validation cohort. Furthermore, ROC curve analysis was used to evaluate the diagnostic value of the gene signature using the “ggpubr” and “pROC” packages.

### 2.6 Functional analysis

Gene Ontology (GO) and Kyoto Encyclopedia of Genes and Genomes (KEGG) analyses were performed to explore the underlying mechanisms on the differentially expressed FRGs. Gene set enrichment analysis (GSEA) was used to select significant pathways associated with each signature gene. Moreover, the CIBERSORT algorithm was used to calculate the relative abundance of 22 types of immune cells in each sample, and the differences between MDS and control samples were compared to explore the potential correlation between MDS and immunity. Spearman correlation analysis was conducted to evaluate the relation between signature genes and immune cells. The outcomes were visualized using the “clusterProfiler,” “enrichplot,” “DOSE,” “pheatmap,” “GSVA,” “GSEABase,” “reshape2,” “ggpubr,” “ggplot2,” and “tidyverse” packages.

### 2.7 Establishment of a competitive endogenous RNA (ceRNA) network

We constructed a ceRNA network by integrating the interactions among mRNAs, microRNAs (miRNAs), and long noncoding RNAs (lncRNAs). The interaction pairs between mRNAs and miRNAs were predicted by intersecting the miRDB, TargetScan, and miRanda databases using signature genes. To identify possible interactions between lncRNAs and miRNAs, we searched the SpongeScan database. The network was visualized using Cytoscape version 3.8.2.

### 2.8 qRT‐PCR

Bone marrow samples were collected from 4 patients with MDS and 4 healthy volunteers. This study received approval from the Ethics Committee of Shanghai Tenth People's Hospital (24K14), and informed consent was obtained from all participants. Peripheral blood mononuclear cells were isolated to validate the expression patterns of the identified genes. Total RNA was extracted using TRIzol reagent (Thermo Fisher Scientific, USA). Subsequently, cDNA was reverse‐transcribed using a PrimeScript^TM^ RT Reagent Kit (Takara, Japan). Quantitative real‐time PCR was carried out with the SYBR Green PCR Master Mix (KAPA, Japan) and the Applied Biosystems 7900HT Fast Real‐Time PCR System (Thermo Fisher Scientific, USA). The relative expression levels of genes were detected using the 2^−ΔΔCt^ method. The primer sequences are listed in Supplementary Table S2. *GAPDH* served as an internal control.

### 2.9 Statistical analysis

Data were analyzed using R 4.1.3 and GraphPad Prism 8.0.1. Student's t‐test was used to compare normally distributed measurement data, whereas Wilcoxon test was used for skewed data. A two‐sided *p‐*value < 0.05 was considered significant.

## 3 RESULTS

### 3.1 Identification of differentially expressed FRGs

**Figure 1:**
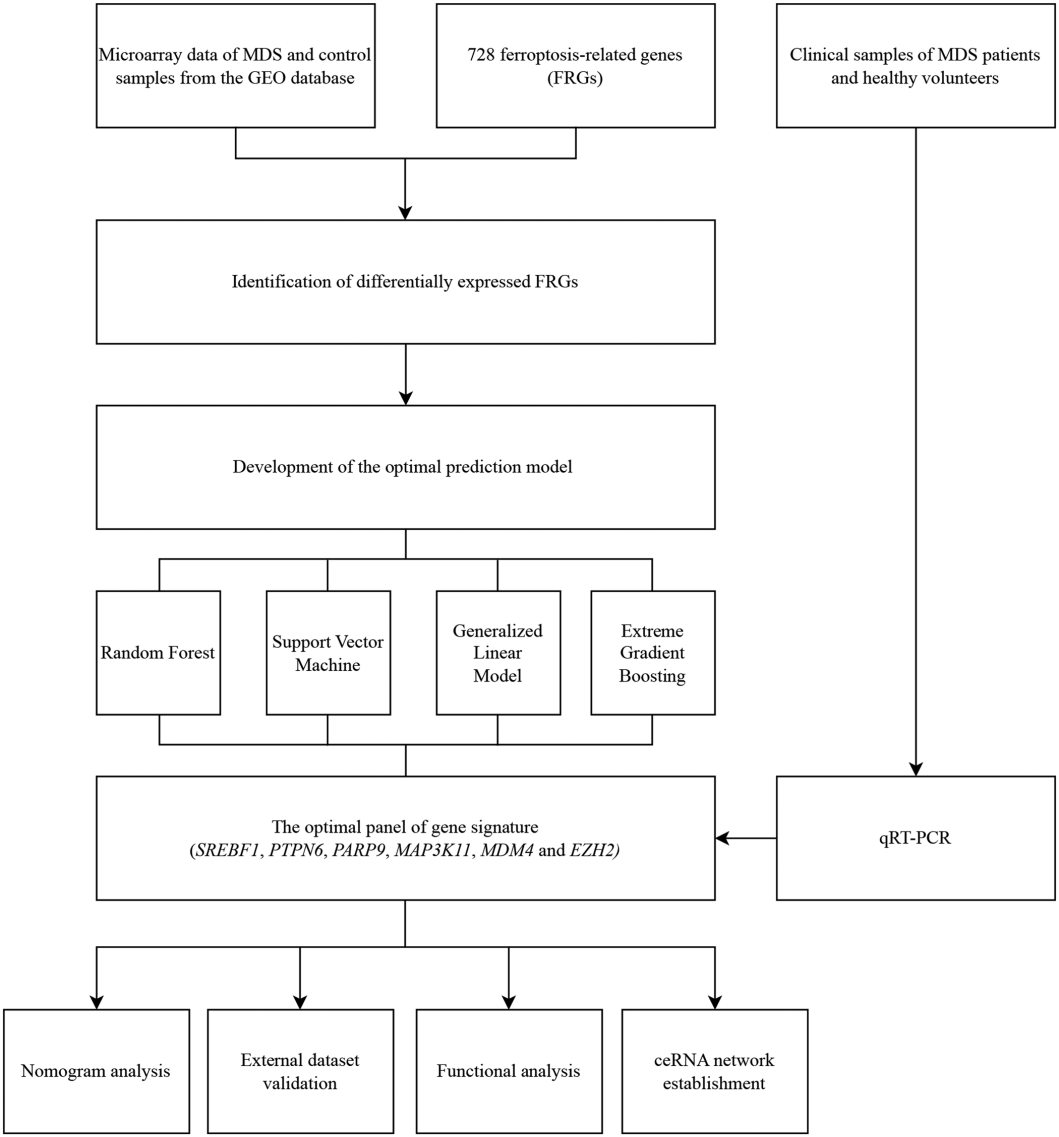
Flowchart of this study. MDS, Myelodysplastic syndromes; qRT‐PCR, Quantitative real‐time polymerase chain reaction; ceRNA, competitive endogenous RNA; GEO, Gene Expression Omnibus.

The flowchart is presented in Figure 1. After analyzing the training set, we identified 117 FRGs as differentially expressed genes between the MDS and control samples. Of these, 61 were upregulated and 56 were downregulated in patients with MDS, meeting the established criteria (Supplementary Table S3).

### 3.2 Establishment of the optimal prediction model

**Figure 2:**
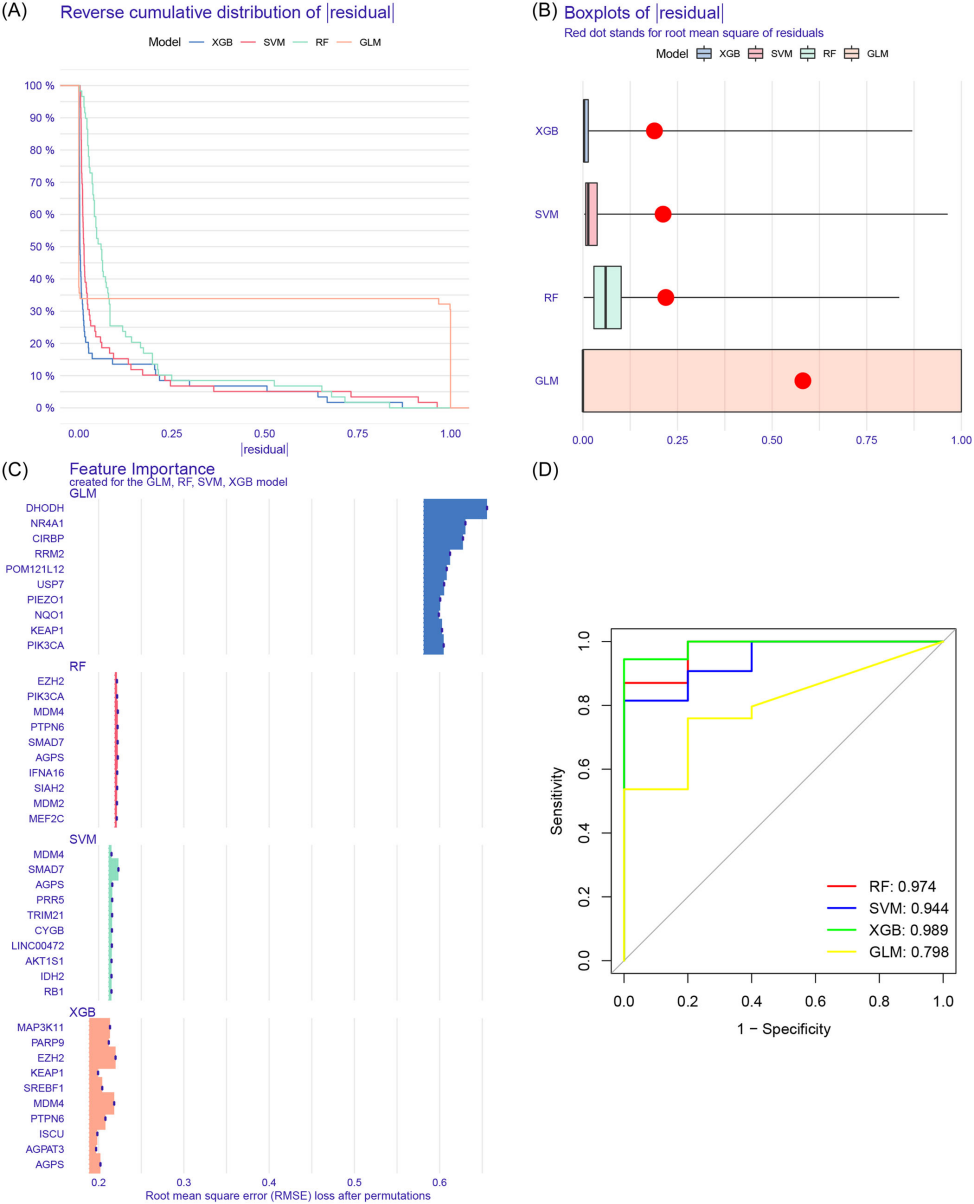
Development of the optimal prediction model. (A) Cumulative residual distribution of each machine learning model, (B) residuals of each machine learning model, (C) important feature variables of each machine learning model, and (D) ROC curves of machine learning models in the training cohort. RF, Random Forest; SVM, Support vector machine learning; XGB, Extreme gradient boosting; GLM, Generalized linear model.

**Figure 3:**
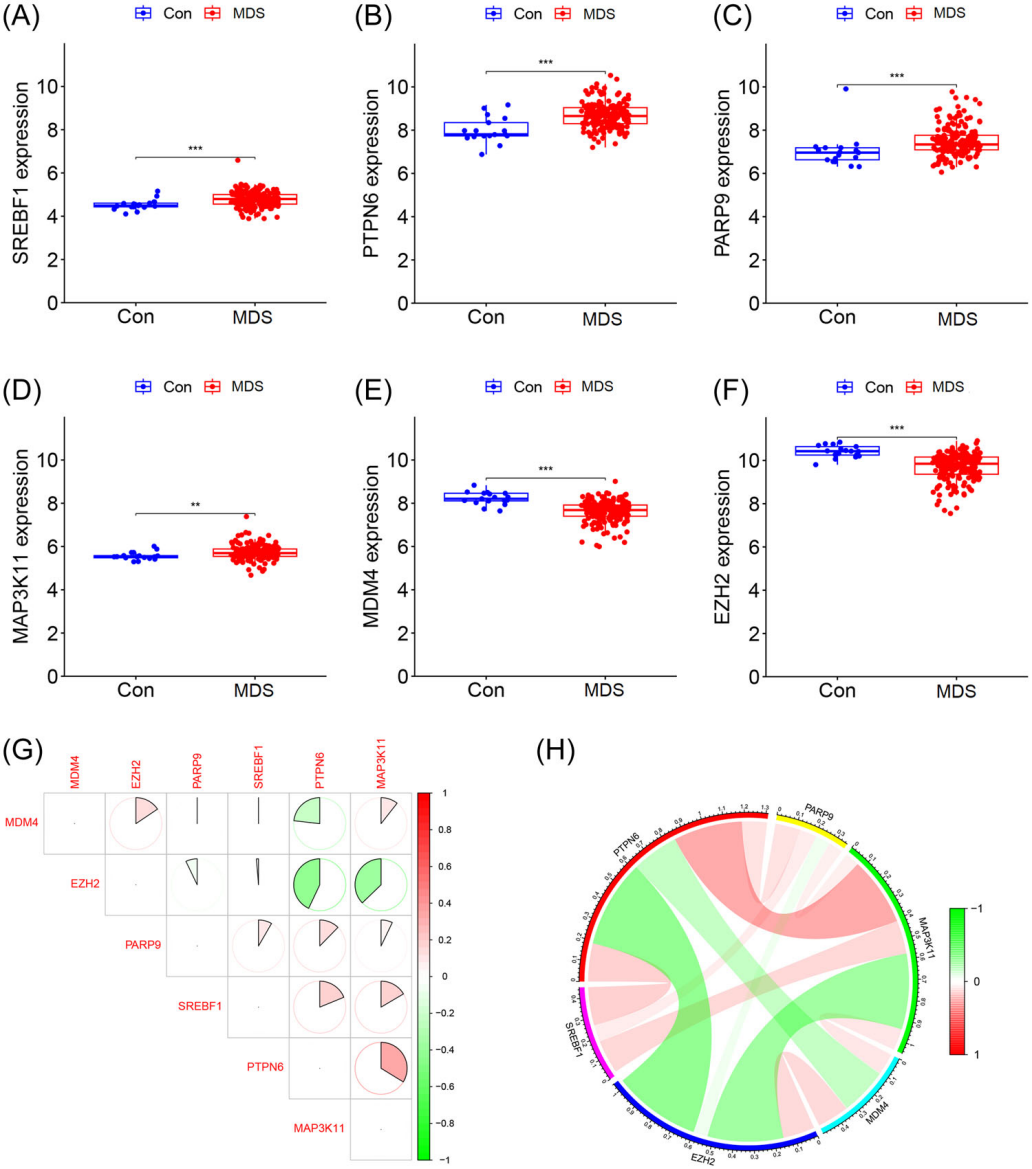
Expression levels of signature genes between MDS and control samples. (A–F) Expression levels of *SREBF1* (A), *PTPN6* (B), *PARP9* (C), *MAP3K11* (D), *MDM4* (E), and *EZH2* (F) in the training cohort. (G) Correlation plot of signature genes. (H) Gene relation network diagram of signature genes. MDS, Myelodysplastic syndromes.

Based on the differentially expressed FRGs, several machine learning models were developed, among which the XGB‐based prediction model exhibited the lowest residuals (Figure 2A and B). In each model, the top 10 feature variables were ranked based on the root mean square error (Figure 2C). The ROC curves demonstrated that the XGB‐based prediction model had the highest area under the curve (AUC) value of 0.989 (Figure 2D). These findings suggested that the XGB algorithm was superior to the other algorithms in diagnosing MDS. Consequently, the top 6 feature variables (*SREBF1*, *PTPN6*, *PARP9*, *MAP3K11*, *MDM4*, and *EZH2*) in the XGB model were considered as the optimal combination of signature genes. In comparison to normal controls, the expression levels of *SREBF1*, *MAP3K11*, *PARP9*, and *PTPN6* were upregulated, whereas *EZH2* and *MDM4* were downregulated in the MDS samples (Figure 3A–F). Moreover, these signature genes showed strong synergistic or antagonistic effects, and their interaction relations were visualized (Figure 3G and H).

### 3.3 Nomogram construction

**Figure 4:**
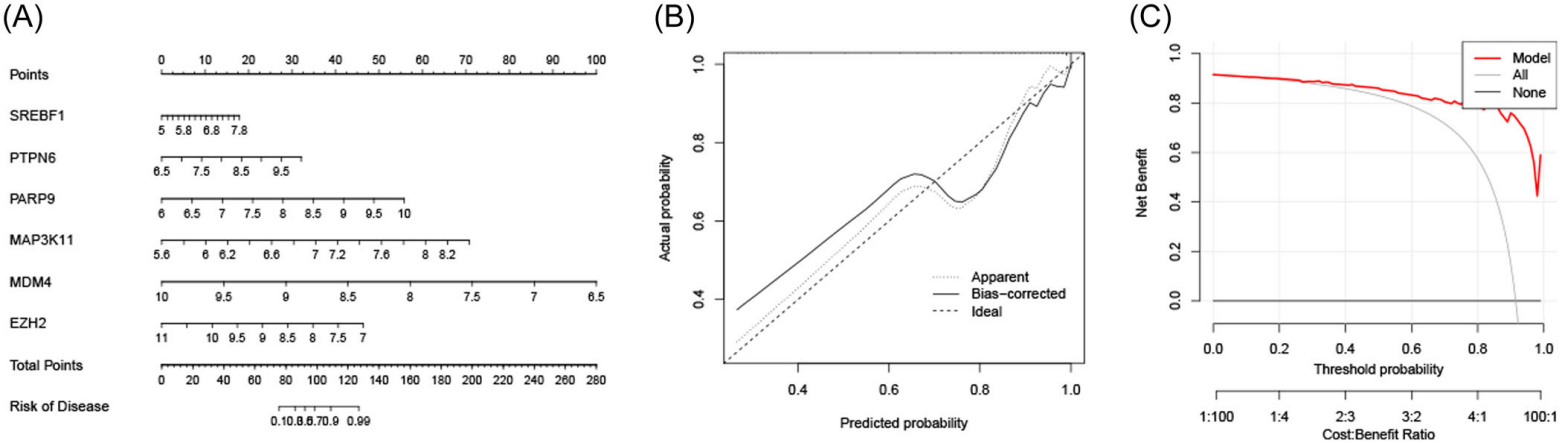
Nomogram construction. (A) Nomogram for predicting the MDS risk, (B) calibration curve, and (C) decision curve analysis. MDS, Myelodysplastic syndromes.

A nomogram was developed to predict the risk of developing MDS (Figure 4A). The calibration curve demonstrated a high degree of concordance between the observed and predicted probabilities (Figure 4B). Furthermore, the decision curve illustrated that the nomogram provided substantial benefits for clinical decision‐making (Figure 4C). These findings indicated that the nomogram model exhibited excellent performance in predicting MDS diagnosis.

### 3.4 External data set validation

**Figure 5:**
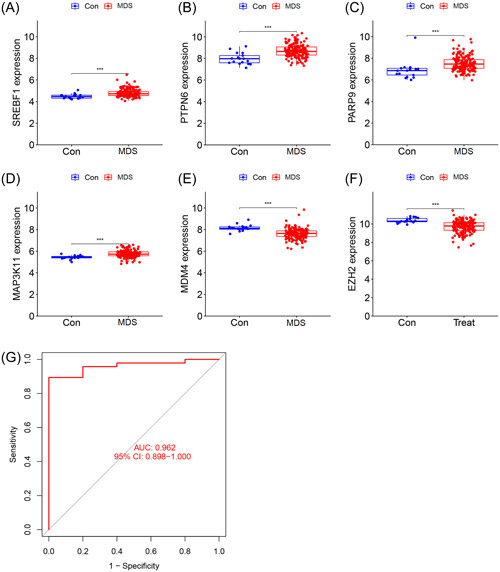
External data set validation. (A–F) Expression levels of *SREBF1* (A), *PTPN6* (B), *PARP9* (C), *MAP3K11* (D), *MDM4* (E), and *EZH2* (F) in MDS and control samples in the validation cohort. (G) ROC curve of the gene signature in the validation cohort. MDS, Myelodysplastic syndromes; ROC, Receiver operating characteristic.

In the independent validation cohort, the signature genes showed gene expression patterns consistent with those in the training cohort **(**Figure 5A–F). The ROC curve also revealed that the gene signature exhibited a high diagnostic value for MDS (AUC = 0.962) (Figure 5G).

### 3.5 Functional analysis

**Figure 6:**
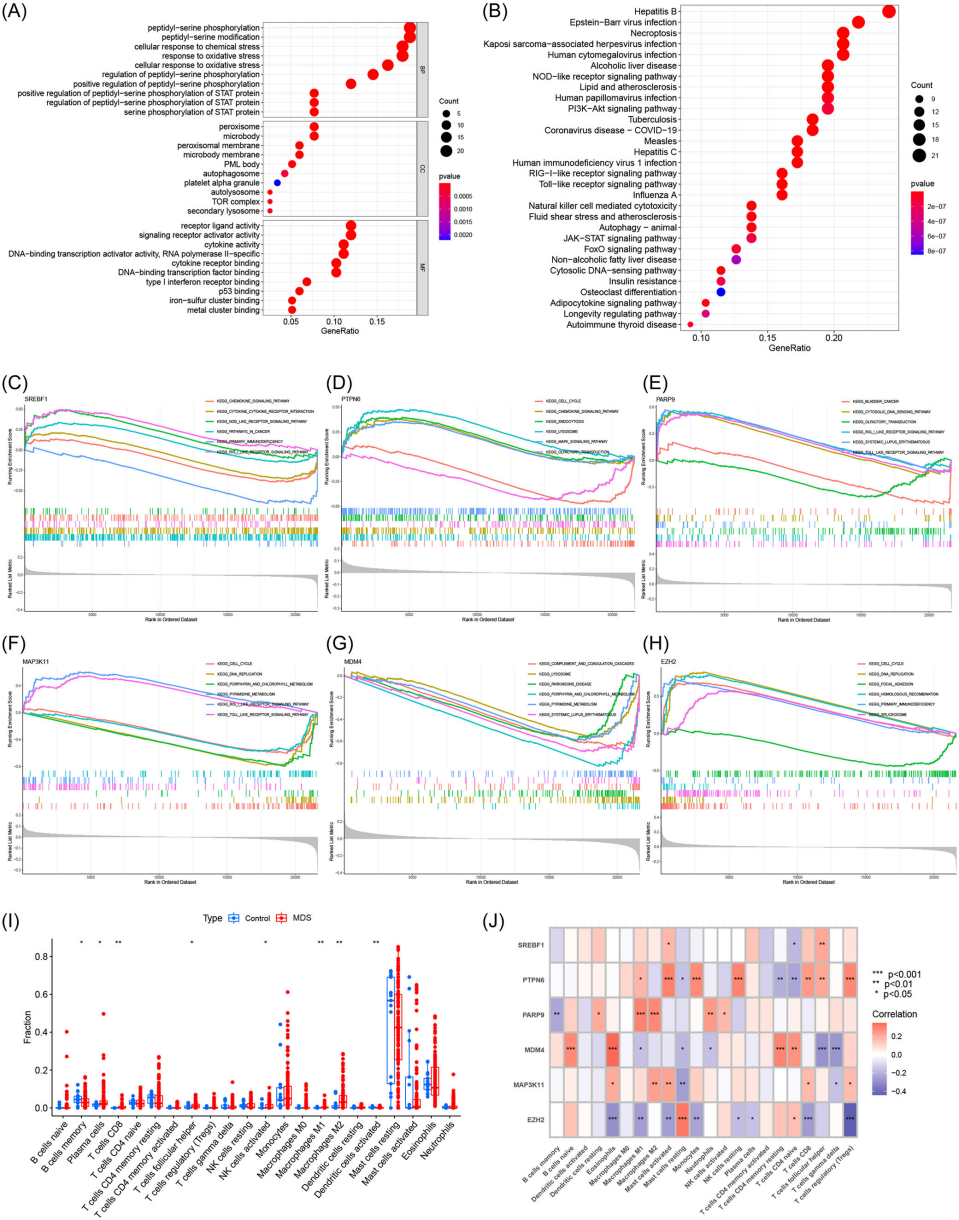
Functional analysis. (A) Bubble diagram of Gene Ontology analysis; (B) bubble diagram of Kyoto Encyclopedia of Genes and Genomes analysis; (C–H) gene set enrichment analysis of the enriched pathways of *SREBF1* (C), *PTPN6* (D), *PARP9* (E), *MAP3K11* (F), *MDM4* (G), and *EZH2* (H). (I) Boxplot of infiltrating immune cells. (J) Heatmap of correlations between genes and infiltrating immune cells. MDS, Myelodysplastic syndromes.

GO analysis revealed a significant enrichment of differentially expressed FRGs in peptidyl‐serine phosphorylation, modification, and cellular responses to chemical stress (Figure 6A). KEGG analysis revealed that these FRGs were significantly involved in immune‐related diseases, such as hepatitis B, Epstein‐Barr virus infection, Kaposi's sarcoma‐associated herpes virus infection, and human cytomegalovirus infection (Figure 6B). Furthermore, GSEA demonstrated that the top 6 pathways enriched by each signature gene were mainly involved in immune‐related pathways, such as the NOD‐like receptor signaling pathway, primary immunodeficiency, and the RIG‐I‐like receptor signaling pathway (Figure 6C–H). In addition, immune cell infiltration analysis showed that memory B cells and activated dendritic cells were significantly lower in MDS samples than in control samples, whereas plasma cells, follicular helper T cells, activated CD8^+^ T cells, activated natural killer cells, and both M1 and M2 macrophages were significantly higher in MDS samples (Figure 6I). Moreover, correlation analysis revealed significant correlations between the signature genes and immune cells (Figure 6J).

### 3.6 Establishment of a ceRNA network

**Figure 7:**
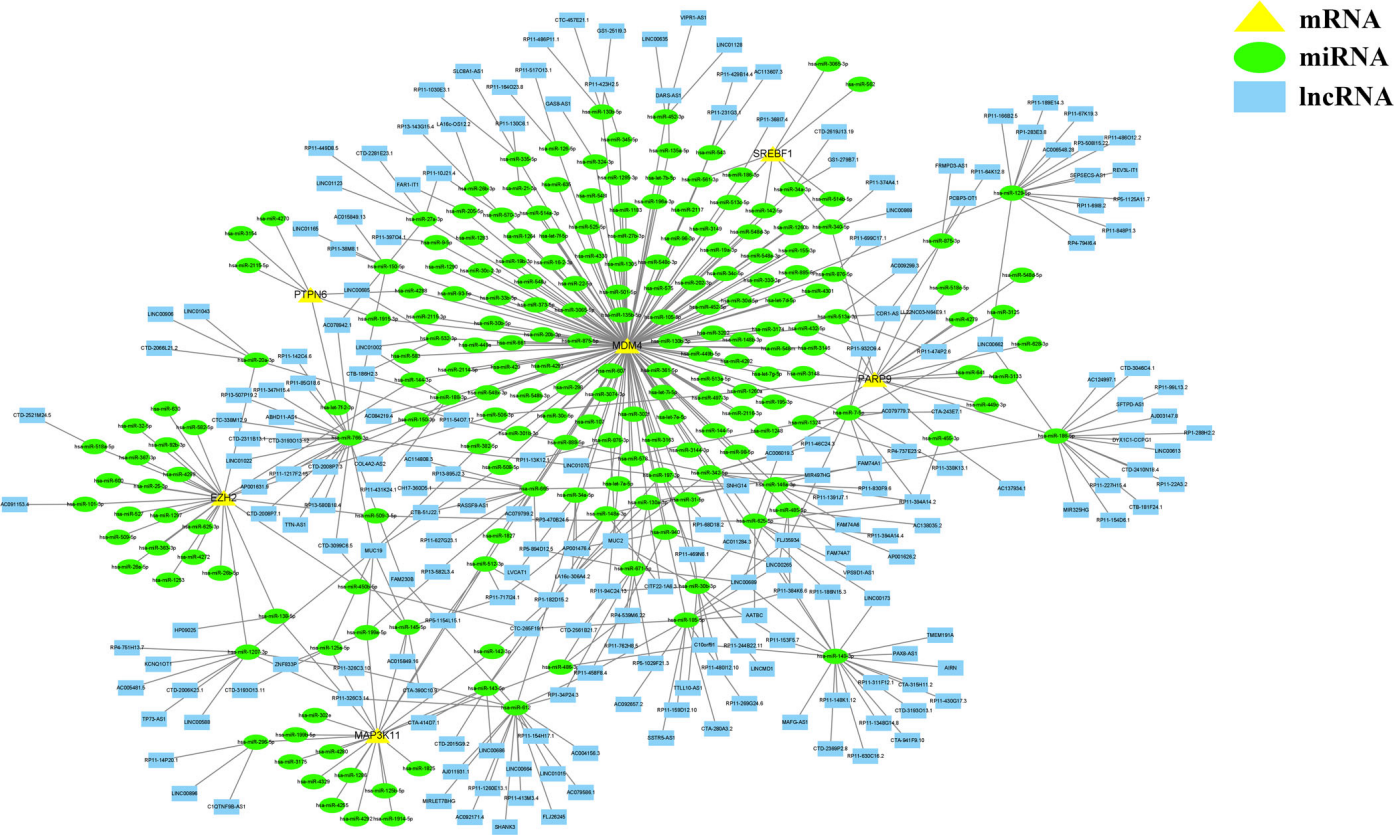
Establishment of a competitive endogenous RNA network. miRNA, MicroRNA; lncRNA, Long noncoding RNA.

To investigate the regulatory mechanisms of the signature genes, a ceRNA network was constructed (Figure 7). This network encompassed 425 nodes, comprising 6 mRNAs, 206 miRNAs, and 213 lncRNAs interconnected by 508 edges (Supplementary Table S4).

### 3.7 qRT‐PCR

**Figure 8:**
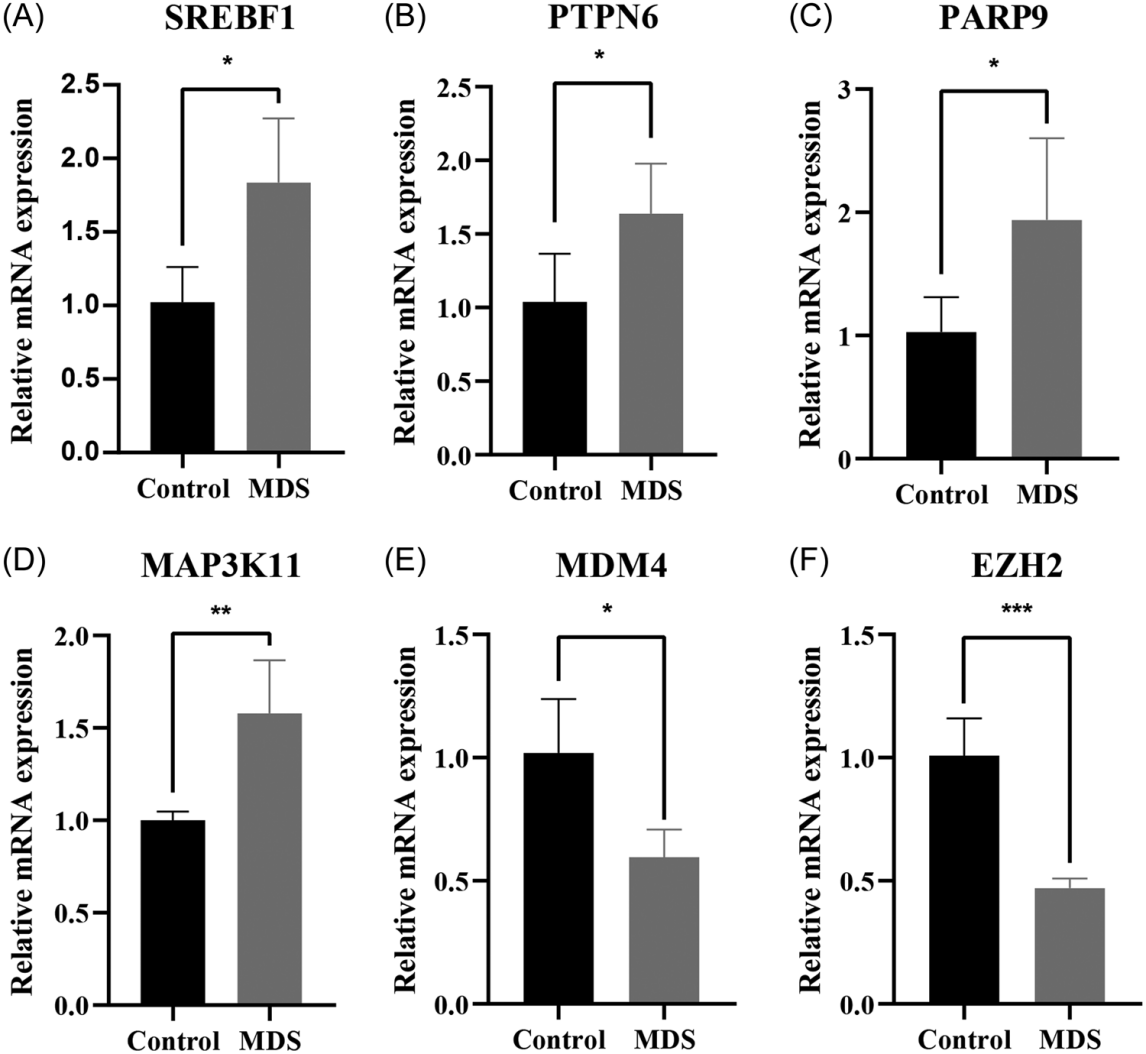
Quantitative real‐time polymerase chain reaction analysis. (A) *SREBF1*; (B) *PTPN6*; (C) *PARP9*; (D) *MAP3K11*; (E) *MDM4*; (F) and *EZH2*. *p  <  0.05; **p  <  0.01; ***p  <  0.001.

Subsequently, qRT‐PCR was employed to validate the expression of the identified genes in both MDS patients and healthy volunteers. As anticipated, compared to healthy volunteers, the expression levels of *SREBF1*, *MAP3K11*, *PARP9*, and *PTPN6* were found to be upregulated, while the expression levels of *EZH2* and *MDM4* were downregulated in the MDS samples (Figure 8).

## 4 DISCUSSION

Given the critical role of prompt and accurate diagnosis coupled with effective MDS management in significantly influencing disease progression and improving overall prognosis, it is essential to investigate the potential effect of FRGs on predicting MDS diagnosis. The key achievement of our study was the pioneering success in constructing and validating a novel signature for patients with MDS based on FRGs. Further analyses revealed a significant correlation between the signature genes and immune‐related pathways. These findings provide a theoretical molecular framework that enhances our understanding of MDS and may inform the development of future diagnostic strategies.

The present study identified 117 differentially expressed FRGs between MDS and control groups, highlighting the crucial role of FRGs in MDS development. Using these differentially expressed FRGs, we constructed prediction models using various machine learning algorithms. Our analysis indicated that the XGB model delivered the most accurate diagnostic predictions for MDS compared with the other tested algorithms. With the highest AUC values and lowest residuals, the XGB model demonstrated superior performance among all the tested models. Consequently, we selected 6 key variables identified using the XGB model to form the optimal signature gene panel: *SREBF1*, *PTPN6*, *PARP9*, *MAP3K11*, *MDM4*, and *EZH2*. In MDS samples, the expression of *SREBF1*, *MAP3K11*, *PARP9*, and *PTPN6* was significantly elevated, while *EZH2* and *MDM4* was notably reduced compared with that in controls. These gene expression patterns were consistent between the training and validation cohorts. It was also worth noting that the gene signature‐based model had AUCs of 0.989 and 0.962 for the 2 cohorts, indicating its robust performance in predicting MDS diagnosis. Among the signature genes, *PTPN6* hypermethylation has been associated with poor prognosis in patients with high‐risk MDS.^40^, ^41^, ^42^ *MDM4* has been implicated as a common mechanism for the transition from preleukemia to acute myeloid leukemia in several genetic disease subtypes.^43^ *EZH2* loss‐of‐function mutations are common in MDS and contribute significantly to its pathogenesis.^44^, ^45^, ^46^ In addition, as a key transcription factor, *SREBF1* regulates genes involved in lipid homeostasis and cholesterol biosynthesis.^47^, ^48^, ^49^ *PARP9* is involved in immune response and DNA damage repair.^50^, ^51^, ^52^ *MAP3K11* activates the JUN N‐terminal pathway and affects microtubule organization during the cell cycle.^53^, ^54^, ^55^ Notably, the expression of these model genes was verified in clinical MDS samples, strengthening the validity of the findings. The exact roles of *SREBF1*, *PARP9*, and *MAP3K11* in MDS remain unclear, and our results provide new insights into the pathogenesis of MDS. Furthermore, the distinct functions of signature genes in MDS warrant further investigation. To the best of our knowledge, there have been some studies on FRG‐based prediction models for human cancers. For example, Shao et al. identified 12 FRGs and generated a prognostic model in acute myeloid leukemia.^56^ Wu et al. established a prognostic prediction model for triple‐negative breast cancer based on 15 FRGs.^57^ Both studies selected gene signatures using LASSO regression. In contrast, we constructed a prediction model using various machine learning algorithms, which increased the reliability of the model.

Functional analysis was performed to investigate the mechanism of action of the signature genes in MDS. KEGG analysis revealed that the differentially expressed FRGs were significantly associated with different types of infection, highlighting their close relation with immunity. GSEA further confirmed this by showing enrichment of signature genes in immune‐related pathways, including the NOD‐like receptor signaling pathway, primary immunodeficiency, and the RIG‐I‐like receptor signaling pathway. Among these pathways, the NOD‐like receptors are intracellular proteins that play a central role in both innate and adaptive immunity.^58^, ^59^ Similarly, the RIG‐I‐like receptors have a significant impact on sensing RNA viral infections and initiating and modulating antiviral immunity.^60^, ^61^ To determine the differences in immune cell infiltration between the 2 groups, the CIBERSORT algorithm was applied and revealed significant differences in the number of infiltrating immune cells between the 2 groups, supporting the notion that immunity plays a key role in MDS progression. The existing literature has increasingly recognized the involvement of different types of immune cells and their downstream pathways in the hematopoietic niche of MDS,^62^, ^63^, ^64^, ^65^ which aligns with our research. Further analysis revealed a significant correlation between the identified genes and immune cells, indicating that these genes may participate in MDS development through immune‐related pathways. Understanding the regulation of these genes by the ceRNA network offers valuable insights into the molecular mechanisms underlying MDS, thereby paving the way for future studies.

Although our prediction model demonstrated satisfactory predictive power and was validated using an external data set, some limitations must be considered. First, our model was established based on available public data. Although qRT‐PCR confirmed the differential gene expression identified in this study, further prospective studies were required to verify its predictive power. Second, the present model did not consider alternative splice forms, which were significantly altered in patients with MDS. Third, the underlying mechanisms linking ferroptosis to MDS progression and the relation between the immune microenvironment and MDS remained unclear. The molecular mechanisms underlying these new biomarkers require further clarification and experimental validation. These elements will be the focus of future research.

In conclusion, our study pioneered the development of a novel 6‐FRG predictive model for patients with MDS. This prediction model demonstrated high diagnostic predictivity with an AUC > 0.9, suggesting that it has a robust potential for effective integration into clinical practice. Furthermore, functional analysis highlighted meaningful associations between the signature genes and immune‐associated pathways. The expression patterns of these genes were consistent in MDS clinical samples, indicating their potential for effective clinical application.

## AUTHOR CONTRIBUTIONS

Yidong Zhu: Conceptualization (lead); writing—original draft (lead); formal analysis (lead). Jun He: Experimental validation (lead). Rong Wei: Writing—review and editing (equal). Jun Liu: Writing—review and editing (equal). All read and authors gave their approval for publication of the final version of the manuscript.

## CONFLICT OF INTEREST STATEMENT

The authors declare no conflict of interest.

## ETHICS APPROVAL AND CONSENT TO PARTICIPATE

This study was approved by the Ethics Committee of Shanghai Tenth People's Hospital (24K14). Informed consent was obtained from all subjects. All methods were performed in accordance with the Declaration of Helsinki.

## CONSENT FOR PUBLICATION

Not applicable.

## Supporting information


**Supplementary Table S1.** Ferroptosis‐related genes.

**Supplementary Table S2.** Primers used for quantitative real‐time polymerase chain reaction.

**Supplementary Table S3.** Identification of differentially expressed ferroptosis‐related genes.

**Supplementary Table S4.** Specific details of the competitive endogenous RNA network.

## Data Availability

The datasets used and/or analyzed during the current study are available from the corresponding author on reasonable request.
