# Supplementary material for: Construction and experimental validation of a novel ferroptosis‐related gene signature for myelodysplastic syndromes
Source: Immun Inflamm Dis. 2024 Apr 5;12(4):e1221. doi: 10.1002/iid3.1221 (PMC10996383; doi:10.1002/iid3.1221)
Supplement: Supplementary file 1 — Supplementary Table S1. Ferroptosis‐related genes. [file IID3-12-e1221-s002.doc]

**Supplementary Table S1. Ferroptosis-related genes.**

| **Ferroptosis-related Genes** |
| --- |
| PTGS2  CHAC1  SLC40A1  TF  TFRC  FTH1  GPX4  HSPB1  NFE2L2  GPX4  FTH1  RPL8  IREB2  ATP5MC3  CS  EMC2  ACSF2  NOX1  CYBB  NOX3  NOX4  NOX5  DUOX1  DUOX2  G6PD  PGD  VDAC2  PIK3CA  FLT3  SCP2  TP53  ACSL4  LPCAT3  NRAS  KRAS  HRAS  TF  TFRC  TFR2  SLC38A1  SLC1A5  GLS2  GOT1  CARS1  TP53  ALOX5  KEAP1  HMOX1  TP53  TP53  GLS2  ATG5  ATG7  NCOA4  TF  ALOX5  ALOX12  ALOX12B  ALOX15  ALOX15B  ALOXE3  PHKG2  TFRC  ACO1  IREB2  SLC38A1  GLS2  G6PDX  ULK1  ATG3  ATG4D  ATG5  BECN1  MAP1LC3A  GABARAPL2  GABARAPL1  ATG16L1  WIPI1  WIPI2  SNX4  ATG13  ULK2  NCOA4  ACSL4  TP53  SAT1  ALOX15  ACSL4  LPCAT3  ALOX15  ACSL4  KEAP1  EGFR  NOX4  MAPK3  MAPK1  BID  ACSL4  ZEB1  KEAP1  DPP4  ALOX15  ALOX12  CDKN2A  PEBP1  SOCS1  CDO1  MYB  HMOX1  MAPK8  MAPK9  MAPK1  MAPK3  SLC1A5  CHAC1  MAPK14  LINC00472  NOX4  GOT1  BECN1  PRKAA2  PRKAA1  ELAVL1  BAP1  TP53  ABCC1  ACSL4  MIR6852  ACVR1B  TGFBR1  BAP1  EPAS1  HILPDA  HIF1A  ALOX12  ACSL4  HMOX1  IFNG  ANO6  LPIN1  HMGB1  TNFAIP3  TLR4  NOX4  ATF3  ATM  YY1AP1  EGLN2  MIOX  TAFAZZIN  MTDH  IDH1  SIRT1  TAFAZZIN  BECN1  FBXW7  PANX1  DNAJB6  BACH1  ACSL4  LONP1  CD82  IL1B  CTSB  POR  CYB5R1  ELOVL5  FADS1  ALOX12  FBXW7  PTEN  NR1D1  NR1D2  TBK1  IL6  USP7  miR-182-5p  miR-378a-3p  CTSB  ACSL4  ATF4  BECN1  AQP3  AQP5  AQP8  LINC00618  IREB2  MT1DP  ACSL4  PEX10  KEAP1  AGPAT3  PEX12  CHP1  GPAT4  BRPF1  OSBPL9  INTS2  MMD  CYP4F8  MLLT1  TTPA  GRIA3  EPT1  POM121L12  LIG3  AEBP2  AGPS  CDCA3  PEX2  LPCAT3  PEX6  TIMM9  DCAF7  LCE2C  FAR1  PHF21A  SMAD7  LYRM1  AMN  PEX3  MTCH1  ZEB1  SIRT1  ACADSB  PVT1  hsa_circ_0008367  SLC39A14  NCOA4  MAP3K11  GSK3B  MAPK8  BRD7  TP53  SLC25A28  ACSL4  MFN2  ACSL4  SLC11A2  ZFAS1  SLC38A1  TSC1  PEBP1  TGFB1  SNCA  SIRT3  PRKAA2  TFRC  CGAS  STING1  HDDC3  MIR761  MDM2  MDM4  ALOX15  POR  MIR214  DLD  LONP1  ACSL4  BACH1  DNAJB6  WWTR1  SIRT1  ATM  PRKCA  LGMN  ACSL4  TP53  IFNG  SMPD1  MYCN  SLC11A2  IFNA1  IFNA2  IFNA4  IFNA5  IFNA6  IFNA7  IFNA8  IFNA10  IFNA13  IFNA14  IFNA16  IFNA17  IFNA21  SMG9  NR1D1  ACSL4  PPARG  TLR4  IL6  MIR335  ATF3  HMOX1  HMGB1  EPAS1  SNX5  PAQR3  MICU1  NOX4  TOR2A  MIR375  MAP3K14  SIRT3  CircKDM4C  MIR324  QSOX1  MIB2  CLTRN  KLF2  MIR5096  TFRC  HOTAIR  H19  FOXO4  ELAVL1  YTHDC2  DDR2  SLC39A7  TRIM46  ACSL1  KDM5A  TRIM21  HMOX1  DPEP1  CYGB  IDO1  GSTZ1  TP53  ACO1  GJA1  IREB2  SLC7A11  PGRMC1  CIRBP  FAR1  circPSEN1  USP11  STING1  YAP1  HMOX1  MIR135B  TRIM26  YAP1  NDRG1  MIR302A  ASMTL-AS1  ZFAS1  FADS2  PIEZO1  LIFR  PTPN6  MIR15A  EGR1  ADAM23  ARHGEF26-AS1  ACSL4  CPEB1  COX4I2  lncRNA AABR07017145.1  TIMP1  MIR15A  KDM6B  NCOA4  GSK3B  IFNG  METTL14  CHAC1  MIB1  KDM5C  ACSL4  MEG3  CCDC6  ATF3  IREB2  CFL1  ALOXE3  MIR539  KMT2D  SLC7A11  GPX4  AKR1C1  AKR1C2  AKR1C3  GPX4  RB1  HSPB1  HSF1  SLC7A11  GPX4  GCLC  SLC7A11  NFE2L2  SQSTM1  NQO1  HMOX1  FTH1  MUC1  SLC3A2  MT1G  NFE2L2  SLC40A1  SLC7A11  GPX4  SLC7A11  CISD1  SLC7A11  FANCD2  GPX4  NFE2L2  FTMT  HSPA5  ATF4  SLC7A11  GPX4  GPX4  HMOX1  ATF4  NFE2L2  TP53  SLC7A11  HELLS  SCD  FADS2  SRC  STAT3  NFE2L2  PML  MTOR  NFS1  TP63  SLC7A11  TP53  CDKN1A  MIR137  SLC40A1  GPX4  GPX4  ENPP2  VDAC2  FH  CISD2  SLC40A1  MIR9-1  MIR9-2  MIR9-3  CBS  NFE2L2  SQSTM1  GPX4  ISCU  FTH1  ACSL3  OTUB1  CD44  LINC00336  STAT3  BRD4  PRDX6  MIR17  SCD  SESN2  NF2  ARNTL  HIF1A  JUN  CA9  HSPA5  TMBIM4  HSPA5  PLIN2  MIR212  Fer1HCH  AIFM2  AIFM2  LAMP2  ZFP36  GPX4  PROM2  CHMP5  CHMP6  AKR1C1  AKR1C2  AKR1C3  CBS  NFE2L2  CAV1  GCH1  SIRT3  DAZAP1  PIR  GCLC  FTL  HCAR1  SLC16A1  RRM2  SCD  NR4A1  PIK3CA  RPTOR  SREBF1  SREBF2  FZD7  NFE2L2  NFE2L2  P4HB  NT5DC2  BCAT2  HSF1  PLA2G6  MIR424  PARK7  FXN  SUV39H1  ATF2  CDKN1A  FTH1  NFE2L2  STAT3  ACOT1  NFE2L2  ALDH3A2  NFE2L2  STK11  FNDC5  CircIL4R  CDH1  NFE2L2  MIR214  NEDD4L  SQSTM1  TF  FTMT  BRD2  BRD3  BRD4  BRDT  SCD  SLC7A11  DECR1  NFE2L2  GPX4  SLC7A11  NFE2L2  GLRX5  GPX4  NCOA3  NR5A2  GPX4  MTOR  PANX2  RHEBP1  TFAP2A  CP  SLC7A11  ARF6  GDF15  ABHD12  PPP1R13L  TFAM  KDM3B  RNF113A  PARK7  AHCY  FXN  circ-TTBK2  MIR522  IDH2  PPARA  NOS2  SIAH2  RELA  PRKAA2  VDR  NEDD4  FXN  AIFM2  PRDX1  AR  CBS  NFE2L2  CHMP5  CHMP6  HMOX1  ZFP36  LAMP2  MTF1  COPZ1  NUPR1  USP35  HSF1  PROM2  PLA2G6  HIF1A  NEAT1  RRM2  SLC7A11  FTMT  PARP1  PARP2  PARP3  PARP4  PARP6  PARP8  PARP9  PARP10  PARP11  PARP12  PARP14  PARP15  PARP16  PDSS2  TXN  SENP1  PLA2G6  OIP5-AS1  MIR190A  FGF21  CREB1  CREB3  CREB5  FTMT  GOT1  TFRC  GPX4  MIR130B  BEX1  ASAH2  SCD  FABP4  AKT1S1  MLST8  MTOR  RPTOR  CDH1  SIRT1  TYRO3  SIRT6  TMSB4X  TMSB4Y  KIF20A  ECH1  circRHOT1  ETV4  MEG8  VCP  circ_0007142  ENPP2  RBMS1  KDM4A  CBS  MGST1  circKIF4A  miR-7-5p  PRDX6  circ_0067934  MPC1  CHMP1A  CAMKK2  SOX2  SRSF9  PROK2  MIR4443  SIRT2  circRNA1615  MIR27A  MIR670  MEF2C  NF2  CDH1  HSPB1  EZH2  PEDS1  SMPD1  ADAMTS13  CDC25A  G6PD  SRSF9  CAV1  CircFNDC3B  PPARD  CISD2  ENO3  SESN2  LCN2  MARCHF5  TRIB2  DHODH  SLC7A11  MIR545  OTUB1  PDK4  CircPVT1  MIR9-3HG  ADIPOQ  circDTL  GPX4  mmu_circRNA_0000309  IL6  PTPN18  FTH1  FTH1  FTL  LCN2  ABCC5  CISD3  MS4A15  LCN2  FURIN  circRHBG  GALNT14  KLHDC3  LINC01833  circGFRA1  MAPKAP1  MLST8  MTOR  PRR5  RICTOR  GSTM1  TERT  circ0097009  TMEM161B-DT  circEPSTI1  MIR18A  RARRES2  USP11 |
