# Supplementary material for: Construction and experimental validation of a novel ferroptosis‐related gene signature for myelodysplastic syndromes
Source: Immun Inflamm Dis. 2024 Apr 5;12(4):e1221. doi: 10.1002/iid3.1221 (PMC10996383; doi:10.1002/iid3.1221)
Supplement: Supplementary file 2 — Supplementary Table S2. Primers used for quantitative real‐time polymerase chain reaction. [file IID3-12-e1221-s001.doc]

**Supplementary Table S2.** Primers used for qRT-PCR.

| Gene name | Strand | 5’ - 3’ |
| --- | --- | --- |
| *SREBF1* | Forward | CCTCCATGGGGTCAGTTGTC |
|  | Reverse | GACTTCTTGCAGGGAGACCC |
| *PARP9* | Forward | AGGTCCTTATGGCTGCCTTT |
|  | Reverse | TTTTGGATCGCAAGGTGTCG |
| *PTPN6* | Forward | TCAAGAACCAGCTGCTAGGC |
|  | Reverse | ATGACACGGCTGTTCTCCTG |
| *MAP3K11* | Forward | CCTGTTGCTGGACCTGGGTAT |
|  | Reverse | GATCAATGCGGCTGCGAAGG |
| *MDM4* | Forward | CCTCAGCACTTGGCAAGGTA |
|  | Reverse | GACGTCAAGACTGGCACTCA |
| *EZH2* | Forward | TTGTGACAGTTCGTGCCCTT |
|  | Reverse | AGCGGCTCCACAAGTAAGAC |
