# Supplementary material for: Construction and experimental validation of a novel ferroptosis‐related gene signature for myelodysplastic syndromes
Source: Immun Inflamm Dis. 2024 Apr 5;12(4):e1221. doi: 10.1002/iid3.1221 (PMC10996383; doi:10.1002/iid3.1221)
Supplement: Supplementary file 3 — Supplementary Table S3. Identification of differentially expressed ferroptosis‐related genes. [file IID3-12-e1221-s004.doc]

**Supplementary Table S3. Identification of differentially expressed FRGs.**

| Gene | conMean | treatMean | pvalue | Type |
| --- | --- | --- | --- | --- |
| IREB2 | 7.45072323529412 | 7.18464545901639 | 0.0132385714841721 | Down |
| PIK3CA | 6.41356488235294 | 6.106982 | 0.0218309273193377 | Down |
| ACSL4 | 5.71225317647059 | 5.50476015846995 | 0.0224687789793545 | Down |
| TFR2 | 6.81551258823529 | 7.18841571038251 | 0.0467194115354913 | Up |
| KEAP1 | 8.46950564705882 | 8.85226231147541 | 0.000773275516350814 | Up |
| ATG5 | 6.87638752941176 | 7.06382036612022 | 0.0280258305805476 | Up |
| ACO1 | 5.449776 | 5.68939914754098 | 0.0138181791672508 | Up |
| MAP1LC3A | 5.01631629411765 | 4.80475232240437 | 0.0403476040380704 | Down |
| MAPK8 | 6.36844105882353 | 6.13288268852459 | 0.00578292563895037 | Down |
| MAPK14 | 7.05817517647059 | 7.27507757377049 | 0.0214557607651924 | Up |
| LINC00472 | 2.73784547058824 | 2.83728475409836 | 0.00073143277361636 | Up |
| PRKAA1 | 6.29722329411765 | 6.06443933333333 | 0.0107855380971555 | Down |
| TGFBR1 | 7.86969417647059 | 7.39842124590164 | 0.00064354408017503 | Down |
| HILPDA | 5.68150511764706 | 5.98254520765027 | 0.0275601805277171 | Up |
| HIF1A | 12.2205072941176 | 11.8528175737705 | 0.00544139555617681 | Down |
| IFNG | 3.03940858823529 | 2.97422161748634 | 0.012524953262099 | Down |
| HMGB1 | 13.0046098823529 | 12.6325563224044 | 6.08983802474713e-07 | Down |
| TNFAIP3 | 9.81989729411765 | 9.08362900546448 | 0.0187607933269152 | Down |
| ATF3 | 6.51243741176471 | 6.00343759562841 | 0.0341692490802113 | Down |
| IDH1 | 8.55762747058824 | 9.00515744262295 | 0.0237938647412067 | Up |
| FBXW7 | 6.57213241176471 | 6.27631827868852 | 0.00189687914950052 | Down |
| PANX1 | 5.84845011764706 | 5.69075444808743 | 0.0212086021197474 | Down |
| LONP1 | 7.81593517647059 | 8.10991896721312 | 0.016172222542792 | Up |
| CD82 | 7.13946676470588 | 7.48432616939891 | 0.0093237699297938 | Up |
| CYB5R1 | 5.285165 | 5.50945212568306 | 0.0220838326145523 | Up |
| NR1D2 | 8.00930547058823 | 7.59227371584699 | 0.00724505500202144 | Down |
| TBK1 | 8.956174 | 9.22248072677596 | 0.00238136949045462 | Up |
| USP7 | 8.58790364705882 | 8.40338213114754 | 0.0182154566170286 | Down |
| AQP3 | 4.74465141176471 | 5.11172236612022 | 0.0107182094242696 | Up |
| AGPAT3 | 6.001444 | 6.28906835519126 | 0.00168396425155464 | Up |
| CHP1 | 7.09239794117647 | 7.29583003278688 | 0.00962562240487254 | Up |
| MMD | 7.23266164705882 | 7.71654348087432 | 0.0259081759858766 | Up |
| EPT1 | 6.861188 | 6.60776066666667 | 0.0152281510422504 | Down |
| POM121L12 | 4.78445129411765 | 4.5338243715847 | 0.0302913500926995 | Down |
| LIG3 | 6.21773541176471 | 5.95213783060109 | 0.00724490217771525 | Down |
| AEBP2 | 7.29245941176471 | 6.99727109836066 | 0.000941578592495399 | Down |
| AGPS | 8.63160711764706 | 8.21596724590164 | 7.33739631227195e-06 | Down |
| PEX6 | 4.69851464705882 | 5.139367 | 0.0414286936522591 | Up |
| TIMM9 | 8.19018464705882 | 8.54481106010929 | 0.000221683369725586 | Up |
| SMAD7 | 6.13675805882353 | 4.24370393442623 | 3.42494051072197e-06 | Down |
| AMN | 4.41658588235294 | 4.58738710382514 | 0.0114824423831745 | Up |
| MAP3K11 | 5.564067 | 5.7283071147541 | 0.0066068456421604 | Up |
| BRD7 | 7.36536411764706 | 7.1641516284153 | 0.00207257780764667 | Down |
| TGFB1 | 4.10234929411765 | 4.55410212021858 | 0.00057016248508974 | Up |
| SNCA | 8.16160876470588 | 8.51731689617486 | 0.0257620572390071 | Up |
| MDM2 | 5.19026035294118 | 4.787747 | 3.61192498426834e-05 | Down |
| MDM4 | 8.22496594117647 | 7.62094612021858 | 2.76843574575096e-07 | Down |
| PRKCA | 3.91845082352941 | 4.26932572131148 | 0.000446208069456173 | Up |
| SMPD1 | 4.29152723529412 | 4.39121547540984 | 0.0166629399727408 | Up |
| IFNA4 | 3.04804664705882 | 2.98747695081967 | 0.0283405528085682 | Down |
| IFNA7 | 3.132428 | 3.03989586338798 | 0.0262019446381038 | Down |
| IFNA10 | 3.05610588235294 | 3.01222131693989 | 0.0251858845650922 | Down |
| IFNA14 | 3.53047452941176 | 3.45017373770492 | 0.0212088445166729 | Down |
| IFNA16 | 3.35550141176471 | 3.24584268306011 | 9.15501873371521e-05 | Down |
| IFNA17 | 4.01918729411765 | 3.80343168306011 | 0.0093238229111521 | Down |
| IFNA21 | 3.23292276470588 | 3.12680009836066 | 0.00548015693970773 | Down |
| PAQR3 | 6.62038688235294 | 6.2193246010929 | 0.00478485101627855 | Down |
| MIB2 | 4.35557952941176 | 4.50773163934426 | 0.0384637074077582 | Up |
| YTHDC2 | 6.647518 | 6.2441662568306 | 0.00176109293051855 | Down |
| ACSL1 | 8.74032358823529 | 8.12259893989071 | 0.0190949929041798 | Down |
| TRIM21 | 5.62334288235294 | 5.93670253005464 | 0.0032417952904466 | Up |
| CYGB | 5.17567611764706 | 4.52331303825137 | 2.13980701593061e-07 | Down |
| SLC7A11 | 3.71898041176471 | 3.98122600546448 | 0.0328920286044568 | Up |
| CIRBP | 9.33772988235294 | 9.1099963989071 | 0.044121715017974 | Down |
| PIEZO1 | 6.64789170588235 | 6.80207755737705 | 0.00956456947169412 | Up |
| PTPN6 | 8.00102129411765 | 8.69529342076503 | 3.68134098429811e-05 | Up |
| ADAM23 | 3.18972782352941 | 3.11192228415301 | 0.00456104645294327 | Down |
| COX4I2 | 5.29717576470588 | 5.10558801092896 | 0.0184322017956412 | Down |
| TIMP1 | 7.59379876470588 | 8.16678693442623 | 0.0188718435569463 | Up |
| MEG3 | 3.91026370588235 | 4.47782892349727 | 0.00345439074835706 | Up |
| RB1 | 8.35172594117647 | 7.96312076502732 | 0.00338212755393449 | Down |
| HSF1 | 5.208152 | 5.45140895081967 | 0.0249018422790744 | Up |
| SQSTM1 | 6.65089217647059 | 7.00232484153005 | 0.0138184637304071 | Up |
| NQO1 | 5.23331870588235 | 5.60092731147541 | 0.0118457044594334 | Up |
| MUC1 | 5.14491170588235 | 5.44306755191257 | 0.0113399466000062 | Up |
| CISD1 | 7.68584788235294 | 8.04702520765027 | 0.0448178428009756 | Up |
| FANCD2 | 6.64204117647059 | 6.27227219672131 | 0.00245158128629273 | Down |
| HELLS | 8.01210664705882 | 7.52142434426229 | 0.00097145758464084 | Down |
| STAT3 | 7.30476964705882 | 7.72941802185792 | 0.000180039623420128 | Up |
| PML | 4.82394352941176 | 4.94834285245902 | 0.0139877942810224 | Up |
| NFS1 | 4.83521647058824 | 5.01751243715847 | 0.0093238229111521 | Up |
| TP63 | 3.20393570588235 | 3.16074839344262 | 0.00285194173617633 | Down |
| ISCU | 10.0011272941176 | 10.3618751311475 | 0.000319717767889784 | Up |
| LAMP2 | 9.10550776470588 | 9.30188278142077 | 0.04575969710768 | Up |
| PROM2 | 4.33047158823529 | 4.18943564480874 | 0.00863337163926638 | Down |
| CHMP6 | 6.28749188235294 | 6.68761792349727 | 0.00665080934839785 | Up |
| PIR | 5.87697117647059 | 6.41164754644809 | 0.0136505945326174 | Up |
| FTL | 13.0859083529412 | 13.5330895300546 | 0.0140580783793002 | Up |
| RRM2 | 10.9534314705882 | 10.2693395628415 | 0.00150435280459623 | Down |
| NR4A1 | 5.79714947058824 | 5.28110904918033 | 0.0242045334232701 | Down |
| SREBF1 | 4.53051535294118 | 4.7846008579235 | 0.000912536652096846 | Up |
| FZD7 | 4.61161847058824 | 5.32789462295082 | 0.000263142145008079 | Up |
| FXN | 5.66111105882353 | 5.92549165027322 | 0.0131575398664876 | Up |
| ALDH3A2 | 7.38488411764706 | 7.72874884153005 | 0.00275158568901134 | Up |
| STK11 | 5.55343617647059 | 5.71524971038251 | 0.00210315160502776 | Up |
| FNDC5 | 3.75740005882353 | 3.65034912021858 | 0.0259081759858766 | Down |
| PANX2 | 4.61359564705882 | 4.4927866557377 | 0.0219571126652886 | Down |
| GDF15 | 3.51245741176471 | 3.94065089071038 | 0.00347875224587086 | Up |
| KDM3B | 8.46145088235294 | 8.14852652459016 | 0.0459982798558694 | Down |
| IDH2 | 9.10974652941177 | 8.80044481967213 | 0.00537027265673143 | Down |
| SIAH2 | 8.74340288235294 | 8.16794450819672 | 0.000160666003147229 | Down |
| RELA | 6.69853964705882 | 6.8975533989071 | 0.0281827837145153 | Up |
| PARP9 | 7.03369782352941 | 7.49631261748634 | 0.000203334849238226 | Up |
| PARP16 | 7.80353776470588 | 7.96483826229508 | 0.0459979536664804 | Up |
| TXN | 9.75190370588235 | 9.93889013114754 | 0.00874457743565222 | Up |
| CREB1 | 8.31811347058824 | 8.05758345355191 | 8.74772976981341e-05 | Down |
| CREB5 | 4.48827417647059 | 4.14599232240437 | 0.0131575741721473 | Down |
| AKT1S1 | 3.053086 | 3.25747878142076 | 0.000407437786687536 | Up |
| TYRO3 | 3.74630964705882 | 3.83476366666667 | 0.0484379038844079 | Up |
| ETV4 | 3.74947376470588 | 3.84445326775956 | 0.00719740454101167 | Up |
| MEF2C | 9.30896611764706 | 8.82011391256831 | 2.16564243697474e-05 | Down |
| EZH2 | 10.4151987647059 | 9.71829430054645 | 4.49774621547419e-07 | Down |
| ENO3 | 4.19212452941176 | 4.36469023497268 | 0.0338000835014979 | Up |
| DHODH | 4.93587964705882 | 5.16638269398907 | 0.018001979818933 | Up |
| PTPN18 | 5.61687882352941 | 5.8263313989071 | 0.0139878301725739 | Up |
| PRR5 | 5.71149576470588 | 6.39488812568306 | 1.36117437063997e-05 | Up |
| TERT | 4.81996323529412 | 4.58198920218579 | 0.00221387289393336 | Down |
