# Supplementary material for: Construction and experimental validation of a novel ferroptosis‐related gene signature for myelodysplastic syndromes
Source: Immun Inflamm Dis. 2024 Apr 5;12(4):e1221. doi: 10.1002/iid3.1221 (PMC10996383; doi:10.1002/iid3.1221)
Supplement: Supplementary file 4 — Supplementary Table S4. Specific details of the competitive endogenous RNA network. [file IID3-12-e1221-s003.doc]

Supplementary Table S4. Specific details of the ceRNA network.

| **miRNA** | **Gene** | **Type** |
| --- | --- | --- |
| hsa-miR-1825 | MAP3K11 | mRNA |
| hsa-miR-340-5p | MDM4 | mRNA |
| hsa-miR-130b-5p | MDM4 | mRNA |
| hsa-miR-3146 | PARP9 | mRNA |
| hsa-miR-107 | MDM4 | mRNA |
| hsa-let-7g-5p | MDM4 | mRNA |
| hsa-miR-671-5p | MDM4 | mRNA |
| hsa-miR-4329 | MAP3K11 | mRNA |
| hsa-miR-1297 | EZH2 | mRNA |
| hsa-miR-548d-5p | PARP9 | mRNA |
| hsa-miR-26b-5p | EZH2 | mRNA |
| hsa-miR-19a-3p | MDM4 | mRNA |
| hsa-miR-570-3p | MDM4 | mRNA |
| hsa-miR-3154 | PTPN6 | mRNA |
| hsa-miR-30b-5p | MDM4 | mRNA |
| hsa-miR-3146 | MDM4 | mRNA |
| hsa-miR-1283 | MDM4 | mRNA |
| hsa-miR-129-5p | MDM4 | mRNA |
| hsa-miR-509-3-5p | EZH2 | mRNA |
| hsa-let-7i-5p | MDM4 | mRNA |
| hsa-miR-525-5p | MDM4 | mRNA |
| hsa-miR-2115-5p | PTPN6 | mRNA |
| hsa-miR-196a-3p | MDM4 | mRNA |
| hsa-miR-514a-3p | MDM4 | mRNA |
| hsa-miR-4301 | MDM4 | mRNA |
| hsa-miR-105-5p | MDM4 | mRNA |
| hsa-miR-324-3p | MDM4 | mRNA |
| hsa-miR-612 | MDM4 | mRNA |
| hsa-miR-130b-3p | MDM4 | mRNA |
| hsa-miR-513a-3p | PARP9 | mRNA |
| hsa-miR-449a | MDM4 | mRNA |
| hsa-miR-4255 | MAP3K11 | mRNA |
| hsa-miR-885-5p | MDM4 | mRNA |
| hsa-miR-2116-3p | MDM4 | mRNA |
| hsa-miR-506-3p | EZH2 | mRNA |
| hsa-miR-1915-3p | MDM4 | mRNA |
| hsa-miR-199b-5p | MAP3K11 | mRNA |
| hsa-miR-561-3p | MDM4 | mRNA |
| hsa-miR-548d-3p | MDM4 | mRNA |
| hsa-miR-452-5p | MDM4 | mRNA |
| hsa-miR-101-3p | EZH2 | mRNA |
| hsa-miR-367-3p | EZH2 | mRNA |
| hsa-miR-32-5p | EZH2 | mRNA |
| hsa-miR-34c-5p | MDM4 | mRNA |
| hsa-miR-625-3p | EZH2 | mRNA |
| hsa-miR-34a-5p | MDM4 | mRNA |
| hsa-miR-455-3p | PARP9 | mRNA |
| hsa-miR-188-3p | MDM4 | mRNA |
| hsa-miR-148a-3p | MDM4 | mRNA |
| hsa-let-7d-5p | MDM4 | mRNA |
| hsa-miR-22-5p | MDM4 | mRNA |
| hsa-miR-888-5p | MDM4 | mRNA |
| hsa-miR-150-3p | EZH2 | mRNA |
| hsa-miR-506-3p | PARP9 | mRNA |
| hsa-miR-31-5p | MDM4 | mRNA |
| hsa-miR-27a-3p | MDM4 | mRNA |
| hsa-miR-3148 | MDM4 | mRNA |
| hsa-miR-20a-3p | EZH2 | mRNA |
| hsa-miR-940 | MDM4 | mRNA |
| hsa-miR-548b-3p | MDM4 | mRNA |
| hsa-let-7f-2-3p | PTPN6 | mRNA |
| hsa-miR-21-3p | MDM4 | mRNA |
| hsa-miR-4280 | MAP3K11 | mRNA |
| hsa-miR-93-5p | MDM4 | mRNA |
| hsa-miR-583 | MDM4 | mRNA |
| hsa-miR-548m | MDM4 | mRNA |
| hsa-miR-661 | MDM4 | mRNA |
| hsa-miR-3174 | MDM4 | mRNA |
| hsa-miR-501-5p | MDM4 | mRNA |
| hsa-miR-3133 | PARP9 | mRNA |
| hsa-miR-30d-5p | MDM4 | mRNA |
| hsa-miR-628-3p | PARP9 | mRNA |
| hsa-miR-96-3p | MDM4 | mRNA |
| hsa-miR-26a-5p | EZH2 | mRNA |
| hsa-miR-543 | MDM4 | mRNA |
| hsa-miR-30c-5p | MDM4 | mRNA |
| hsa-miR-186-3p | MDM4 | mRNA |
| hsa-miR-146a-3p | MDM4 | mRNA |
| hsa-miR-19b-3p | MDM4 | mRNA |
| hsa-miR-607 | MDM4 | mRNA |
| hsa-miR-518a-5p | EZH2 | mRNA |
| hsa-miR-34a-3p | MDM4 | mRNA |
| hsa-miR-195-3p | MDM4 | mRNA |
| hsa-miR-138-5p | EZH2 | mRNA |
| hsa-miR-301b-3p | MDM4 | mRNA |
| hsa-miR-2114-5p | MDM4 | mRNA |
| hsa-miR-1264 | MDM4 | mRNA |
| hsa-miR-125a-5p | MAP3K11 | mRNA |
| hsa-miR-298 | MDM4 | mRNA |
| hsa-miR-512-3p | MAP3K11 | mRNA |
| hsa-miR-452-3p | MDM4 | mRNA |
| hsa-miR-27b-3p | MDM4 | mRNA |
| hsa-miR-600 | EZH2 | mRNA |
| hsa-miR-432-5p | MDM4 | mRNA |
| hsa-miR-4330 | MDM4 | mRNA |
| hsa-miR-485-5p | MDM4 | mRNA |
| hsa-miR-143-5p | MAP3K11 | mRNA |
| hsa-miR-449b-5p | MDM4 | mRNA |
| hsa-miR-144-3p | EZH2 | mRNA |
| hsa-miR-1285-3p | MDM4 | mRNA |
| hsa-miR-202-3p | MDM4 | mRNA |
| hsa-miR-1827 | MDM4 | mRNA |
| hsa-miR-25-3p | EZH2 | mRNA |
| hsa-miR-512-3p | MDM4 | mRNA |
| hsa-let-7b-5p | MDM4 | mRNA |
| hsa-miR-185-5p | MDM4 | mRNA |
| hsa-miR-561-3p | SREBF1 | mRNA |
| hsa-miR-30b-3p | MDM4 | mRNA |
| hsa-miR-4288 | MDM4 | mRNA |
| hsa-miR-135b-5p | MDM4 | mRNA |
| hsa-miR-345-5p | MDM4 | mRNA |
| hsa-miR-1207-3p | EZH2 | mRNA |
| hsa-miR-145-5p | MAP3K11 | mRNA |
| hsa-miR-635 | MDM4 | mRNA |
| hsa-miR-548c-3p | MDM4 | mRNA |
| hsa-miR-363-3p | EZH2 | mRNA |
| hsa-miR-532-3p | MDM4 | mRNA |
| hsa-miR-509-5p | EZH2 | mRNA |
| hsa-miR-144-5p | MDM4 | mRNA |
| hsa-miR-302e | MAP3K11 | mRNA |
| hsa-miR-296-5p | MAP3K11 | mRNA |
| hsa-miR-513a-3p | MDM4 | mRNA |
| hsa-miR-2115-3p | MDM4 | mRNA |
| hsa-miR-514b-5p | SREBF1 | mRNA |
| hsa-miR-197-3p | MDM4 | mRNA |
| hsa-miR-766-3p | MDM4 | mRNA |
| hsa-miR-1260a | MDM4 | mRNA |
| hsa-miR-1915-3p | PTPN6 | mRNA |
| hsa-miR-4297 | MDM4 | mRNA |
| hsa-miR-1324 | PARP9 | mRNA |
| hsa-miR-205-5p | MDM4 | mRNA |
| hsa-miR-30c-2-3p | MDM4 | mRNA |
| hsa-miR-135a-5p | MDM4 | mRNA |
| hsa-miR-4292 | MAP3K11 | mRNA |
| hsa-miR-665 | MDM4 | mRNA |
| hsa-miR-527 | EZH2 | mRNA |
| hsa-miR-1183 | MDM4 | mRNA |
| hsa-miR-630 | EZH2 | mRNA |
| hsa-miR-4272 | EZH2 | mRNA |
| hsa-miR-340-5p | SREBF1 | mRNA |
| hsa-miR-513a-5p | MDM4 | mRNA |
| hsa-miR-3065-5p | MDM4 | mRNA |
| hsa-miR-149-3p | MDM4 | mRNA |
| hsa-miR-497-3p | MDM4 | mRNA |
| hsa-miR-4279 | PARP9 | mRNA |
| hsa-miR-562 | SREBF1 | mRNA |
| hsa-miR-125b-5p | MAP3K11 | mRNA |
| hsa-miR-150-3p | MDM4 | mRNA |
| hsa-miR-548l | MDM4 | mRNA |
| hsa-miR-1248 | MDM4 | mRNA |
| hsa-miR-1286 | MAP3K11 | mRNA |
| hsa-miR-1253 | EZH2 | mRNA |
| hsa-miR-3065-3p | SREBF1 | mRNA |
| hsa-miR-548a-3p | MDM4 | mRNA |
| hsa-miR-3202 | MDM4 | mRNA |
| hsa-miR-142-5p | SREBF1 | mRNA |
| hsa-miR-3149 | MDM4 | mRNA |
| hsa-miR-302f | MDM4 | mRNA |
| hsa-miR-3148 | PARP9 | mRNA |
| hsa-miR-513c-5p | MDM4 | mRNA |
| hsa-let-7a-5p | MDM4 | mRNA |
| hsa-miR-3175 | MAP3K11 | mRNA |
| hsa-miR-876-5p | MDM4 | mRNA |
| hsa-miR-508-5p | MDM4 | mRNA |
| hsa-miR-1827 | MAP3K11 | mRNA |
| hsa-miR-575 | MDM4 | mRNA |
| hsa-miR-3125 | PARP9 | mRNA |
| hsa-miR-548u | MDM4 | mRNA |
| hsa-miR-142-3p | MAP3K11 | mRNA |
| hsa-miR-3074-3p | MDM4 | mRNA |
| hsa-miR-1305 | MDM4 | mRNA |
| hsa-miR-361-5p | MDM4 | mRNA |
| hsa-miR-186-5p | PARP9 | mRNA |
| hsa-miR-148b-3p | MDM4 | mRNA |
| hsa-miR-126-5p | MDM4 | mRNA |
| hsa-miR-429 | MDM4 | mRNA |
| hsa-miR-876-3p | MDM4 | mRNA |
| hsa-miR-625-5p | MDM4 | mRNA |
| hsa-miR-450b-5p | EZH2 | mRNA |
| hsa-let-7f-5p | MDM4 | mRNA |
| hsa-miR-4299 | EZH2 | mRNA |
| hsa-miR-150-5p | MDM4 | mRNA |
| hsa-miR-138-5p | MAP3K11 | mRNA |
| hsa-miR-548x-3p | MDM4 | mRNA |
| hsa-miR-2117 | MDM4 | mRNA |
| hsa-miR-16-2-3p | MDM4 | mRNA |
| hsa-miR-199a-5p | MAP3K11 | mRNA |
| hsa-miR-98-5p | MDM4 | mRNA |
| hsa-miR-155-3p | MDM4 | mRNA |
| hsa-miR-7-5p | MDM4 | mRNA |
| hsa-miR-449c-3p | PARP9 | mRNA |
| hsa-miR-340-5p | PARP9 | mRNA |
| hsa-miR-513c-5p | SREBF1 | mRNA |
| hsa-miR-875-3p | PARP9 | mRNA |
| hsa-miR-1260b | MDM4 | mRNA |
| hsa-miR-4288 | PTPN6 | mRNA |
| hsa-miR-514b-5p | MDM4 | mRNA |
| hsa-miR-26b-3p | MDM4 | mRNA |
| hsa-miR-92b-3p | EZH2 | mRNA |
| hsa-let-7e-5p | MDM4 | mRNA |
| hsa-miR-641 | PARP9 | mRNA |
| hsa-miR-578 | MDM4 | mRNA |
| hsa-miR-875-5p | MDM4 | mRNA |
| hsa-miR-9-5p | MDM4 | mRNA |
| hsa-miR-342-5p | MDM4 | mRNA |
| hsa-miR-373-5p | MDM4 | mRNA |
| hsa-miR-486-3p | MAP3K11 | mRNA |
| hsa-miR-130a-3p | MDM4 | mRNA |
| hsa-miR-382-5p | MDM4 | mRNA |
| hsa-miR-20b-3p | MDM4 | mRNA |
| hsa-miR-335-5p | MDM4 | mRNA |
| hsa-miR-144-3p | MDM4 | mRNA |
| hsa-miR-582-5p | EZH2 | mRNA |
| hsa-miR-3144-3p | MDM4 | mRNA |
| hsa-miR-1914-5p | MAP3K11 | mRNA |
| hsa-miR-33b-5p | MDM4 | mRNA |
| hsa-miR-330-3p | MDM4 | mRNA |
| hsa-miR-142-5p | MDM4 | mRNA |
| hsa-miR-1290 | MDM4 | mRNA |
| hsa-miR-3163 | MDM4 | mRNA |
| hsa-miR-4282 | MDM4 | mRNA |
| hsa-miR-4270 | PTPN6 | mRNA |
| hsa-miR-518c-5p | PARP9 | mRNA |
| hsa-miR-7-5p | CDR1-AS | lncRNA |
| hsa-miR-612 | RP11-326C3.10 | lncRNA |
| hsa-miR-570-3p | RP11-10J21.4 | lncRNA |
| hsa-miR-143-5p | CTA-414D7.1 | lncRNA |
| hsa-miR-1207-3p | MUC19 | lncRNA |
| hsa-miR-875-3p | CDR1-AS | lncRNA |
| hsa-miR-7-5p | RP11-830F9.6 | lncRNA |
| hsa-miR-27a-3p | RP11-10J21.4 | lncRNA |
| hsa-miR-671-5p | RP5-894D12.5 | lncRNA |
| hsa-miR-665 | RP5-894D12.5 | lncRNA |
| hsa-miR-612 | RP1-34P24.3 | lncRNA |
| hsa-miR-671-5p | RP1-34P24.3 | lncRNA |
| hsa-miR-197-3p | LA16c-306A4.2 | lncRNA |
| hsa-miR-31-5p | C10orf91 | lncRNA |
| hsa-miR-30b-3p | C10orf91 | lncRNA |
| hsa-miR-149-3p | C10orf91 | lncRNA |
| hsa-miR-145-5p | MUC19 | lncRNA |
| hsa-miR-1324 | AC079779.7 | lncRNA |
| hsa-miR-26b-3p | LA16c-OS12.2 | lncRNA |
| hsa-miR-143-5p | CTC-265F19.1 | lncRNA |
| hsa-miR-612 | RP11-326C3.14 | lncRNA |
| hsa-miR-876-5p | CDR1-AS | lncRNA |
| hsa-miR-148a-3p | CITF22-1A6.3 | lncRNA |
| hsa-miR-130a-3p | CITF22-1A6.3 | lncRNA |
| hsa-miR-7-5p | FLJ35934 | lncRNA |
| hsa-miR-146a-3p | FAM74A1 | lncRNA |
| hsa-miR-146a-3p | RP11-830F9.6 | lncRNA |
| hsa-miR-612 | FAM230B | lncRNA |
| hsa-miR-486-3p | C10orf91 | lncRNA |
| hsa-miR-665 | RP11-627G23.1 | lncRNA |
| hsa-miR-185-5p | CTC-265F19.1 | lncRNA |
| hsa-miR-766-3p | LINC01070 | lncRNA |
| hsa-miR-138-5p | HP09025 | lncRNA |
| hsa-miR-185-5p | AC092657.2 | lncRNA |
| hsa-miR-186-5p | RP11-99L13.2 | lncRNA |
| hsa-miR-188-3p | CTB-186H2.3 | lncRNA |
| hsa-miR-296-5p | RP11-14P20.1 | lncRNA |
| hsa-miR-1207-3p | RP11-326C3.10 | lncRNA |
| hsa-miR-197-3p | LINC01070 | lncRNA |
| hsa-miR-146a-3p | FAM74A7 | lncRNA |
| hsa-miR-612 | RP11-458F8.4 | lncRNA |
| hsa-miR-1207-3p | RP11-326C3.14 | lncRNA |
| hsa-miR-146a-3p | FAM74A6 | lncRNA |
| hsa-miR-1207-3p | TP73-AS1 | lncRNA |
| hsa-miR-101-3p | AC091153.4 | lncRNA |
| hsa-miR-185-5p | AATBC | lncRNA |
| hsa-miR-766-3p | RP13-507P19.2 | lncRNA |
| hsa-miR-148a-3p | LA16c-306A4.2 | lncRNA |
| hsa-miR-34a-5p | LA16c-306A4.2 | lncRNA |
| hsa-miR-130a-3p | LA16c-306A4.2 | lncRNA |
| hsa-miR-150-5p | LINC01002 | lncRNA |
| hsa-miR-188-3p | LINC01002 | lncRNA |
| hsa-miR-34a-5p | AP001476.4 | lncRNA |
| hsa-miR-543 | RP11-429B14.4 | lncRNA |
| hsa-miR-186-5p | MIR325HG | lncRNA |
| hsa-miR-7-5p | RP11-338K13.1 | lncRNA |
| hsa-miR-20a-3p | LINC01043 | lncRNA |
| hsa-miR-188-3p | COL4A2-AS2 | lncRNA |
| hsa-miR-625-5p | RP11-394A14.2 | lncRNA |
| hsa-miR-512-3p | RP11-717I24.1 | lncRNA |
| hsa-miR-508-5p | MUC19 | lncRNA |
| hsa-miR-7-5p | RP11-932O9.4 | lncRNA |
| hsa-miR-766-3p | MUC19 | lncRNA |
| hsa-miR-1324 | RP4-737E23.2 | lncRNA |
| hsa-miR-129-5p | RP11-166B2.5 | lncRNA |
| hsa-miR-149-3p | CTA-941F9.10 | lncRNA |
| hsa-miR-452-3p | VIPR1-AS1 | lncRNA |
| hsa-miR-450b-5p | CTC-265F19.1 | lncRNA |
| hsa-miR-766-3p | CTD-2008P7.3 | lncRNA |
| hsa-miR-30b-3p | MUC2 | lncRNA |
| hsa-miR-625-5p | AATBC | lncRNA |
| hsa-miR-612 | RP11-1260E13.1 | lncRNA |
| hsa-miR-766-3p | RP13-580B18.4 | lncRNA |
| hsa-miR-143-5p | LA16c-306A4.2 | lncRNA |
| hsa-miR-625-5p | RP3-470B24.5 | lncRNA |
| hsa-miR-150-5p | LINC01165 | lncRNA |
| hsa-miR-149-3p | AIRN | lncRNA |
| hsa-miR-188-3p | AC084219.4 | lncRNA |
| hsa-miR-766-3p | AC078942.1 | lncRNA |
| hsa-miR-342-5p | RP11-46C24.3 | lncRNA |
| hsa-miR-875-3p | PCBP3-OT1 | lncRNA |
| hsa-miR-665 | CTB-51J22.1 | lncRNA |
| hsa-let-7a-5p | RP11-94C24.13 | lncRNA |
| hsa-miR-486-3p | RP11-94C24.13 | lncRNA |
| hsa-miR-148a-3p | RP11-717I24.1 | lncRNA |
| hsa-miR-185-5p | RP11-384K6.6 | lncRNA |
| hsa-miR-671-5p | CTD-2561B21.7 | lncRNA |
| hsa-miR-7-5p | AC006019.3 | lncRNA |
| hsa-miR-512-3p | RP1-182D15.2 | lncRNA |
| hsa-miR-185-5p | FLJ35934 | lncRNA |
| hsa-miR-561-3p | RP11-231G3.1 | lncRNA |
| hsa-miR-142-3p | MUC2 | lncRNA |
| hsa-miR-625-5p | LINC00173 | lncRNA |
| hsa-miR-20a-3p | RP11-54O7.17 | lncRNA |
| hsa-miR-149-3p | LINC00265 | lncRNA |
| hsa-miR-149-3p | RP11-311F12.1 | lncRNA |
| hsa-miR-766-3p | RP11-1217F2.15 | lncRNA |
| hsa-miR-130b-5p | GS1-251I9.3 | lncRNA |
| hsa-miR-766-3p | CTD-3193O13.12 | lncRNA |
| hsa-miR-149-3p | LINC00689 | lncRNA |
| hsa-miR-129-5p | AC006548.28 | lncRNA |
| hsa-miR-145-5p | CTD-3099C6.5 | lncRNA |
| hsa-miR-342-5p | AC011284.3 | lncRNA |
| hsa-miR-148a-3p | RP1-182D15.2 | lncRNA |
| hsa-miR-148a-3p | SNHG14 | lncRNA |
| hsa-miR-130a-3p | SNHG14 | lncRNA |
| hsa-miR-186-5p | LINC00613 | lncRNA |
| hsa-miR-199a-5p | MUC19 | lncRNA |
| hsa-miR-671-5p | RP5-1029F21.3 | lncRNA |
| hsa-miR-185-5p | RP11-269G24.6 | lncRNA |
| hsa-miR-342-5p | MUC2 | lncRNA |
| hsa-miR-455-3p | AC137934.1 | lncRNA |
| hsa-miR-126-5p | RP11-164O23.8 | lncRNA |
| hsa-miR-199a-5p | ZNF833P | lncRNA |
| hsa-miR-671-5p | RP11-762H8.5 | lncRNA |
| hsa-miR-27a-3p | RP11-449D8.5 | lncRNA |
| hsa-miR-766-3p | RP11-85G18.6 | lncRNA |
| hsa-miR-382-5p | MUC19 | lncRNA |
| hsa-miR-342-5p | RP11-394A14.2 | lncRNA |
| hsa-miR-30b-3p | LINCMD1 | lncRNA |
| hsa-miR-186-5p | DYX1C1-CCPG1 | lncRNA |
| hsa-miR-30b-3p | RP11-153F5.7 | lncRNA |
| hsa-miR-625-5p | AC006019.3 | lncRNA |
| hsa-miR-612 | AC004156.3 | lncRNA |
| hsa-miR-766-3p | LINC01022 | lncRNA |
| hsa-miR-452-3p | LINC01128 | lncRNA |
| hsa-miR-345-5p | RP11-423H2.5 | lncRNA |
| hsa-miR-875-3p | RP11-64K12.8 | lncRNA |
| hsa-miR-1207-3p | CTD-2006K23.1 | lncRNA |
| hsa-miR-875-3p | FRMPD3-AS1 | lncRNA |
| hsa-miR-30b-3p | AC011284.3 | lncRNA |
| hsa-miR-186-5p | RP11-154D6.1 | lncRNA |
| hsa-miR-766-3p | RP11-142C4.6 | lncRNA |
| hsa-miR-27a-3p | LINC01123 | lncRNA |
| hsa-miR-185-5p | CTA-280A3.2 | lncRNA |
| hsa-miR-766-3p | LINC01002 | lncRNA |
| hsa-miR-149-3p | CTA-315H11.2 | lncRNA |
| hsa-miR-143-5p | LINC00686 | lncRNA |
| hsa-miR-149-3p | RP11-153F5.7 | lncRNA |
| hsa-miR-149-3p | LINC00173 | lncRNA |
| hsa-miR-665 | RP13-895J2.3 | lncRNA |
| hsa-miR-342-5p | RP11-1391J7.1 | lncRNA |
| hsa-miR-185-5p | RP11-159D12.10 | lncRNA |
| hsa-miR-186-5p | RP11-22A3.2 | lncRNA |
| hsa-miR-1207-3p | KCNQ1OT1 | lncRNA |
| hsa-miR-26b-3p | RP13-143G15.4 | lncRNA |
| hsa-miR-185-5p | LINC00265 | lncRNA |
| hsa-miR-186-5p | SFTPD-AS1 | lncRNA |
| hsa-miR-342-5p | RP11-54O7.17 | lncRNA |
| hsa-miR-150-5p | AC015849.13 | lncRNA |
| hsa-miR-149-3p | TMEM191A | lncRNA |
| hsa-miR-1207-3p | LINC00588 | lncRNA |
| hsa-miR-143-5p | RP11-154H17.1 | lncRNA |
| hsa-miR-149-3p | RP11-186N15.3 | lncRNA |
| hsa-miR-612 | LINC00664 | lncRNA |
| hsa-miR-665 | RP13-582L3.4 | lncRNA |
| hsa-miR-129-5p | LINC00662 | lncRNA |
| hsa-miR-625-5p | RP11-186N15.3 | lncRNA |
| hsa-miR-146a-3p | LINC00689 | lncRNA |
| hsa-miR-1324 | LINC01070 | lncRNA |
| hsa-miR-197-3p | RP11-469N6.1 | lncRNA |
| hsa-miR-7-5p | RP11-394A14.2 | lncRNA |
| hsa-miR-205-5p | FAR1-IT1 | lncRNA |
| hsa-miR-612 | MIRLET7BHG | lncRNA |
| hsa-miR-625-5p | VPS9D1-AS1 | lncRNA |
| hsa-miR-197-3p | SNHG14 | lncRNA |
| hsa-miR-20a-3p | LINC00906 | lncRNA |
| hsa-miR-766-3p | CTD-2311B13.1 | lncRNA |
| hsa-miR-145-5p | CTA-390C10.9 | lncRNA |
| hsa-miR-150-5p | RP11-38M8.1 | lncRNA |
| hsa-miR-766-3p | CTC-338M12.9 | lncRNA |
| hsa-miR-543 | AC113607.3 | lncRNA |
| hsa-miR-129-5p | RP11-69I8.2 | lncRNA |
| hsa-miR-455-3p | RP11-394A14.2 | lncRNA |
| hsa-miR-197-3p | RP1-68D18.2 | lncRNA |
| hsa-miR-513a-3p | RP11-474P2.6 | lncRNA |
| hsa-miR-125a-5p | MUC19 | lncRNA |
| hsa-miR-30b-3p | TTLL10-AS1 | lncRNA |
| hsa-miR-665 | SNHG14 | lncRNA |
| hsa-miR-766-3p | AP001631.9 | lncRNA |
| hsa-miR-149-3p | CTD-3193O13.1 | lncRNA |
| hsa-miR-665 | CH17-360D5.1 | lncRNA |
| hsa-miR-766-3p | TTN-AS1 | lncRNA |
| hsa-miR-7-5p | LINC00662 | lncRNA |
| hsa-miR-30b-3p | RP11-94C24.13 | lncRNA |
| hsa-miR-34a-3p | CTD-2619J13.19 | lncRNA |
| hsa-miR-34a-3p | GS1-279B7.1 | lncRNA |
| hsa-miR-342-5p | MIR497HG | lncRNA |
| hsa-miR-186-5p | RP1-288H2.2 | lncRNA |
| hsa-miR-27a-3p | CTD-2281E23.1 | lncRNA |
| hsa-miR-21-3p | RP11-130C6.1 | lncRNA |
| hsa-miR-888-5p | AC079799.2 | lncRNA |
| hsa-miR-188-3p | RP11-431K24.1 | lncRNA |
| hsa-miR-485-5p | AP001626.2 | lncRNA |
| hsa-miR-149-3p | MAFG-AS1 | lncRNA |
| hsa-miR-145-5p | RP11-717I24.1 | lncRNA |
| hsa-miR-432-5p | RP11-932O9.4 | lncRNA |
| hsa-miR-130a-3p | RP4-539M6.22 | lncRNA |
| hsa-miR-1207-3p | AC005481.5 | lncRNA |
| hsa-miR-296-5p | LINC00896 | lncRNA |
| hsa-miR-671-5p | LINC00689 | lncRNA |
| hsa-miR-612 | LINC01015 | lncRNA |
| hsa-miR-143-5p | CTD-2015G9.2 | lncRNA |
| hsa-miR-135a-5p | DARS-AS1 | lncRNA |
| hsa-miR-876-5p | RP11-699C17.1 | lncRNA |
| hsa-miR-129-5p | RP11-67K19.3 | lncRNA |
| hsa-miR-512-3p | RP5-1154L15.1 | lncRNA |
| hsa-miR-186-5p | AC124997.1 | lncRNA |
| hsa-miR-129-5p | REV3L-IT1 | lncRNA |
| hsa-miR-612 | AJ011931.1 | lncRNA |
| hsa-miR-665 | AC114808.3 | lncRNA |
| hsa-miR-145-5p | AC015849.16 | lncRNA |
| hsa-miR-30b-3p | RP11-244B22.11 | lncRNA |
| hsa-miR-612 | RP11-413M3.4 | lncRNA |
| hsa-miR-485-5p | LINC00265 | lncRNA |
| hsa-miR-766-3p | CTD-3099C6.5 | lncRNA |
| hsa-miR-149-3p | RP11-148K1.12 | lncRNA |
| hsa-miR-625-5p | RP11-394A14.4 | lncRNA |
| hsa-miR-335-5p | SLC8A1-AS1 | lncRNA |
| hsa-miR-129-5p | RP5-1125A11.7 | lncRNA |
| hsa-miR-340-5p | LINC00869 | lncRNA |
| hsa-miR-342-5p | AC006019.3 | lncRNA |
| hsa-miR-125a-5p | CTD-3193O13.11 | lncRNA |
| hsa-miR-186-5p | CTD-2410N18.4 | lncRNA |
| hsa-miR-149-3p | RP11-430G17.3 | lncRNA |
| hsa-miR-129-5p | RP3-508I15.22 | lncRNA |
| hsa-miR-126-5p | RP11-517O13.1 | lncRNA |
| hsa-miR-149-3p | RP11-1348G14.8 | lncRNA |
| hsa-miR-186-5p | AJ003147.8 | lncRNA |
| hsa-miR-296-5p | C1QTNF9B-AS1 | lncRNA |
| hsa-miR-129-5p | SEPSECS-AS1 | lncRNA |
| hsa-miR-34a-5p | LVCAT1 | lncRNA |
| hsa-miR-9-5p | RP11-397O4.1 | lncRNA |
| hsa-miR-186-5p | CTD-3046C4.1 | lncRNA |
| hsa-miR-186-5p | RP11-227H15.4 | lncRNA |
| hsa-miR-149-3p | PAX8-AS1 | lncRNA |
| hsa-miR-766-3p | CTD-2008P7.1 | lncRNA |
| hsa-miR-513a-3p | LL22NC03-N64E9.1 | lncRNA |
| hsa-miR-612 | FLJ26245 | lncRNA |
| hsa-miR-185-5p | SSTR5-AS1 | lncRNA |
| hsa-miR-612 | AC079586.1 | lncRNA |
| hsa-miR-612 | SHANK3 | lncRNA |
| hsa-miR-612 | AC092171.4 | lncRNA |
| hsa-miR-129-5p | RP11-848P1.3 | lncRNA |
| hsa-miR-186-5p | LINC00662 | lncRNA |
| hsa-miR-129-5p | RP11-486O12.2 | lncRNA |
| hsa-miR-518a-5p | CTD-2521M24.5 | lncRNA |
| hsa-miR-30b-3p | RP11-480I12.10 | lncRNA |
| hsa-miR-186-5p | CTB-181F24.1 | lncRNA |
| hsa-miR-665 | RASSF8-AS1 | lncRNA |
| hsa-miR-1207-3p | RP4-751H13.7 | lncRNA |
| hsa-miR-199a-5p | RP13-582L3.4 | lncRNA |
| hsa-miR-485-5p | RP11-384K6.6 | lncRNA |
| hsa-miR-129-5p | RP1-283E3.8 | lncRNA |
| hsa-miR-149-3p | RP11-630C16.2 | lncRNA |
| hsa-miR-766-3p | ABHD11-AS1 | lncRNA |
| hsa-miR-130b-5p | RP11-486P11.1 | lncRNA |
| hsa-miR-7-5p | CTA-243E7.1 | lncRNA |
| hsa-miR-129-5p | RP11-189E14.3 | lncRNA |
| hsa-miR-486-3p | RP4-539M6.22 | lncRNA |
| hsa-miR-513a-3p | AC009299.3 | lncRNA |
| hsa-miR-485-5p | AC138035.2 | lncRNA |
| hsa-miR-340-5p | RP11-374A4.1 | lncRNA |
| hsa-miR-27a-3p | AC078942.1 | lncRNA |
| hsa-miR-185-5p | RP11-458F8.4 | lncRNA |
| hsa-miR-452-3p | LINC00635 | lncRNA |
| hsa-miR-186-3p | RP11-368I7.4 | lncRNA |
| hsa-miR-149-3p | CTD-2369P2.8 | lncRNA |
| hsa-miR-335-5p | RP11-1030E3.1 | lncRNA |
| hsa-miR-20a-3p | CTD-2066L21.2 | lncRNA |
| hsa-miR-130b-5p | CTC-457E21.1 | lncRNA |
| hsa-miR-129-5p | RP4-794I6.4 | lncRNA |
| hsa-miR-186-5p | SNHG14 | lncRNA |
| hsa-miR-876-3p | RP11-13K12.1 | lncRNA |
| hsa-miR-876-3p | MUC2 | lncRNA |
| hsa-miR-532-3p | CTB-186H2.3 | lncRNA |
| hsa-miR-532-3p | LINC01002 | lncRNA |
| hsa-miR-324-3p | GAS8-AS1 | lncRNA |
| hsa-miR-940 | RP11-458F8.4 | lncRNA |
| hsa-miR-509-3-5p | RP3-470B24.5 | lncRNA |
| hsa-miR-766-3p | RP11-347H15.4 | lncRNA |
| hsa-let-7f-2-3p | FAM230B | lncRNA |
| hsa-miR-532-3p | LINC00685 | lncRNA |
| hsa-miR-940 | AP001476.4 | lncRNA |
| hsa-miR-940 | LINC00265 | lncRNA |
